# Is apical periodontitis a matter of microbial diversity or time? A scoping review

**DOI:** 10.1186/s13005-026-00610-4

**Published:** 2026-03-31

**Authors:** Sally Mohamed Nazir, Samar Mahmoud M. Saied, Mark Ezzat Eskander, Mai M. Mansour, Hams Abdelrahman, Rania Mahmoud Elbackly, Hisham Mahmoud Elnawam

**Affiliations:** 1 Endodontics, Conservative Dentistry Department, Faculty of Dentistry, Alexandria University, Champolion St., Azarita, Alexandria, 21527 Egypt; 2 Tissue Engineering Laboratories, Faculty of Dentistry, Alexandria University, Champolion St., Azarita, Alexandria, 21527 Egypt; 3 Pediatric Dentistry and Dental Public health Department, Faculty of Dentistry, Alexandria University, Champolion St., Azarita, Alexandria, 21527 Egypt

**Keywords:** Apical periodontitis, Periapical lesion, Microbial diversity, Endodontic infections, Endodontic microbiology

## Abstract

**Background:**

The pathophysiology of apical periodontitis is complicated involving host immunological response, virulence factors, and a diverse microbiome. Understanding how microbial diversity influences lesion size is crucial for improving therapeutic strategies.

**Objective:**

This scoping review aimed to have an insight through literature to determine whether primary or secondary apical periodontitis lesions of different sizes are correlated with the quantity and diversity of microorganisms or the duration of the disease.

**Methods:**

The Joanna Briggs Institute (JBI) methodology for conducting scoping reviews was followed. A comprehensive electronic search was conducted through PubMed, Scopus, Web of Science, and Google Scholar to identify relevant studies published up to February 2025. In addition, handsearching was performed to identify additional studies that were not retrieved in the electronic search. Eligibility criteria of the screened papers included clinical studies performed in healthy patients with symptomatic or asymptomatic apical periodontitis where microbial analysis was performed. The EndNote Web reference manager (EndNote X9; Thomson Reuters) was used to ingest articles from various sources, categorize the references, and automatically eliminate duplicates.

**Results:**

Out of 4010 papers, 132 studies met the inclusion requirements and were added to the current review. Approximately half of the papers examined bacterial diversity in endodontic infections, while just a small percentage discussed lesion size and were identified as randomized clinical trials. Brazil, USA, Germany and China were found to have the highest frequency of published articles. Fusobacteria, Streptococcus, Enterococcus faecalis and Porphyromonas species were the most detected microorganisms responsible for apical pathosis regardless of lesion size, while other microbiomes were associated with large lesions only, such as Olsenella, Lactococcus lactus and HHV-6 with 3% each and HPV with 6.1%. Other microbiomes such as Candida albicans, Filifactor alocis, HSV1, Pyramidobacter piscolens and Phocaeicola abscessus were seen only associated with small sized lesions with 4.3% each.

**Conclusions:**

Microbial diversity and microbial load seem to be a strong determinant of apical lesion size while lesion duration could not be adequately assessed due to cross-sectional study designs. Lesion size is an important variant to be recorded to give insight into microbial diversity and provide the basis for personalized targeted antimicrobial therapies in the future.

This scoping review was registered in the open science framework; DOI: 10.17605/OSF.IO/DC95Z.

**Supplementary Information:**

The online version contains supplementary material available at 10.1186/s13005-026-00610-4.

## Introduction

Apical periodontitis is an inflammatory condition occurring due to endodontic infections either due to primary infections resulting from pulpal necrosis that allows bacterial by-products to diffuse into periapical tissues, inducing a host immune response for repair, or due to secondary infection as a result of endodontic treatment failure or persistent infection [1]. These lesions typically manifest radiographically as radiolucencies around the tooth apex, encompassing granulomas, cysts, and abscesses, and may persist or recur following initial endodontic treatment [2].

Emerging evidence suggests that microbial diversity within apical lesions may influence their progression and size [3, 4]. It could also involve key pathogen causing the disease, or it could be a time factor for these microbial communities to mutually thrive and induce bone loss causing larger more resistant lesions [3, 4]. Apical periodontitis has also been linked to viruses, fungi, and archaea [5, 6]. But in endodontic infections, bacteria continue to be the most prevalent and powerful pathogens [3, 4].

The primary form of cellular bacteria found in nature is represented by biofilms contributing to their resistant to antimicrobial treatment [7]. According to Ricucci and Siqueira, apical periodontitis is reported to be a biofilm-mediated disease and that there is a close association between bacterial biofilms and primary and post-treatment apical periodontitis [8].

Recent microbial detection methods including culture-independent analysis using bacterial ribosomal RNA (rRNA) genes have provided valuable insights into the diverse microbial communities in the root canals [9, 10]. Several endodontic infections that can lead to lesion formation and impede healing have lately been linked to newly identified species and phylotypes that were not previously cultivated [11]. This emphasizes the need to characterize microbial community composition in relation to disease severity [11].

Although studies have investigated the nature and specificity of different microbial species contributing to endodontic infections [12], there is high interindividual case to case Variability in species number and composition within the endodontic microbiome [8, 9].

The accurate diagnosis of periapical lesions is ideally performed by histopathological examination; however, this is unfeasible in cases of non-surgical endodontic treatment of lesions of different sizes [10]. Therefore, lesion size is commonly measured using periapical radiographs or cone-beam computed tomography (CBCT) which shows promising results in evaluation of periapical condition [10]. This is often used as an indicator of infection severity and helps guide treatment decisions [11]. A recent study has shown that in primary endodontic infections, root canal volume determined by CBCT can give an estimation of bacterial counts [12]. Although CBCT is more sensitive than traditional two-dimensional (2D) X-rays in detection of apical lesions, studies have shown that this higher detection does not always lead to altered clinical decisions [13]. Even so, using standardized radiographic protocols remains essential to ensure accurate lesion measurement and consistency between studies [14].

Endodontic literature has often addressed the relationship between lesion size and the duration of the periapical infection. Studies have shown that larger periapical lesions are often associated with long standing infections and higher microbial load. However, the clear interrelationship between the size of periapical lesions and microbial diversity within the context of time is unclear. Hence, this review aimed to map the literature in search for a clear answer of whether it is microbial diversity or the duration of infection or both that has a direct influence on the size of periapical lesions which would inevitably have a consequence on the dynamics of healing post endodontic treatment or retreatment [3, 4].This question is clinically relevant from an endodontic standpoint as it may influence diagnostic interpretation and prognosis following endodontic treatment and retreatment. Given the heterogeneity in microbial sampling methods and radiographic evaluation criteria, a Joanna Briggs Institute (JBI)-compliant scoping review [15, 16] was conducted to map, explore and chart the current available studies that assess the relationship between root canal microbial diversity and radiographically determined lesion size in systemically healthy patients undergoing primary or secondary endodontic therapy to identify gaps, and clarify concept outlines.

## Methods

This scoping review was conducted in accordance with the Joanna Briggs Institute (JBI) Methodology for Scoping Reviews [16] and registered in the open science framework (OPF) under the number 10.17605/OSF.IO/DC95Z. The focused PCC question was: Is it microbial diversity or lesion duration that affects the size of periapical lesions in endodontic infection? Where P (Population) represents patients with periapical lesions related to primary, persistent/secondary endodontic infection; C (Concept) represents microbial diversity; C (Context) represents periapical lesions with different sizes.

### Literature search, study selection and screening

A search of the electronic literature was done with PubMed, Web of Science, Scopus and google scholar till February 2025, in addition to articles found relevant by hand search. The search strategy adopted for each database is presented in Table 1.

Search results from the databases were exported to EndNote (EndNote X9; Thomson Reuters, New York, NY, EUA), for duplicates removal, then all articles were exported to an Excel spreadsheet. Four reviewers (SN, SS, ME, MM) independently screened the records based on their titles and abstracts. A second stage, performed by the same reviewers, consisted of reading the full-text manuscripts of the potentially eligible studies to assess their eligibility. Disagreements on studies’ eligibility were solved by consensus with a fifth reviewer (HE).

**Table 1:** Search strategy and initial results This table summarizes the search terms used, and the number of initial results retrieved from each database included in this scoping review

Database	Search Terms Used	Initial Results Retrieved
PubMed	#1 (((((((((((“Periapical Periodontitis“[Mesh]) OR (“Periapical Abscess“[Mesh]))) OR (“Periapical Granuloma“[Mesh])) OR (asymptomatic apical periodontitis)) OR (symptomatic apical periodontitis)) OR (chronic apical periodontitis)) OR (post treatment apical periodontitis)) OR (root canal retreatment)) OR (endodontic infection)) OR (intra radicular infection)) OR (extra radicular infection)) AND (((((((((((((((((((((((((bacterial species) OR (“Microbiota“[Mesh])) OR (microbiome)) OR (resistant bacteria)) OR (Microbial communities)) OR (Microbiological profile)) OR (“Enterococcus faecalis“[Mesh])) OR (“Fusobacterium“[Mesh])) OR (“Porphyromonas gingivalis“[Mesh])) OR (herpes virus)) OR (phyla)) OR (taxa)) OR (“Polymerase Chain Reaction“[Mesh])) OR (molecular methods)) OR (“Phylogeny“[Mesh])) OR (16 S ribosomal RNA sequencing)) OR (colony forming unit)) OR (DNA sequence)) OR (“DNA Sequence, Unstable“[Mesh])) OR (“RNA, Ribosomal, 16S“[Mesh])) OR (“Mass Spectrometry“[Mesh])) OR (molecular identification)) OR (taxonomy units)) AND (“Endodontics“[Mesh])) OR (“Root Canal Therapy“[Mesh]))) AND ((((“Cone-Beam Computed Tomography“[Mesh) OR (periapical radiograph)) OR (cone beam periapical index)) OR (periapical index))	13,783
	#2 (((((((((((“Periapical Periodontitis“[Mesh]) OR (“Periapical Abscess“[Mesh]))) OR (“Periapical Granuloma“[Mesh])) OR (asymptomatic apical periodontitis)) OR (symptomatic apical periodontitis)) OR (chronic apical periodontitis)) OR (post treatment apical periodontitis)) OR (root canal retreatment)) OR (endodontic infection)) OR (intra radicular infection)) OR (extra radicular infection)	22,197
	#3 (“Cone-Beam Computed Tomography“[Mesh) OR (periapical radiograph)) OR (cone beam periapical index)) OR (periapical index) AND ((((((((((((((((((((((((bacterial species) OR (“Microbiota“[Mesh])) OR (microbiome)) OR (resistant bacteria)) OR (Microbial communities)) OR (Microbiological profile)) OR (“Enterococcus faecalis“[Mesh])) OR (“Fusobacterium“[Mesh])) OR (“Porphyromonas gingivalis“[Mesh])) OR (herpes virus)) OR (phyla)) OR (taxa)) OR (“Polymerase Chain Reaction“[Mesh])) OR (molecular methods)) OR (“Phylogeny“[Mesh])) OR (16 S ribosomal RNA sequencing)) OR (colony forming unit)) OR (DNA sequence)) OR (“DNA Sequence, Unstable“[Mesh])) OR (“RNA, Ribosomal, 16S“[Mesh])) OR (“Mass Spectrometry“[Mesh])) OR (molecular identification)) OR (taxonomy units)) AND (“Endodontics“[Mesh])) OR (“Root Canal Therapy“[Mesh])	23,981
	#1 AND #2 AND #3	1038
Scopus	#1(ALL (microbiomes) OR ALL (microbiota) OR ALL (molecular AND identification) OR ALL (polymerase AND chain AND reaction) OR ALL (16 s AND ribosomal AND rna) AND ALL (endodontics)) #2(ALL (cone AND beam AND computed AND tomography) OR ALL (periapical AND radiograph) AND ALL (endodontics) OR ALL (periapical AND lesion)) **#3**(ALL (apical AND periodontitis) OR ALL (post AND treatment AND apical AND periodontitis) OR ALL (endodontic AND infection)) AND ((ALL (apical AND periodontitis) OR ALL (post AND treatment AND apical AND periodontitis) OR ALL (endodontic AND infection)))	22,382 18,936 37,965
	#1 AND #2 AND #3	480
Web of Science	#1 ((((((((((((((((ALL=(secondary periapical infection)) OR ALL=(endodontic infection)) OR ALL=(intra radicular infection)) OR ALL=(extra radicular infection)) OR ALL=(Root Canal Therap*)) OR ALL=(root canal treatment)) OR ALL=(retreatment)) OR ALL=(post treatment periodontitis)) OR ALL=(periodontitis)) OR ALL=(Chronic)) OR ALL=(Nonsuppurative)) OR ALL=(Periapical Abscess)) OR ALL=(Suppurative Periapical Periodontitis)) OR ALL=(Granuloma*)) OR ALL=(chronic apical periodontitis)) OR ALL=(primary periapical infection)) OR ALL=(refractory apical periodontitis) #2 (((((ALL=(cone beam computed tomography)) OR ALL=(periapical radiograph)) OR ALL=(periapical index)) OR ALL=(radiographic)) OR ALL=(x ray)) #3 ((((((((((((((((((((((((((ALL=(bacterial species)) OR ALL=(Microbio*)) OR ALL=(resistant bacteria)) OR ALL=(Microbial communit*)) OR ALL=(Microbiological profile)) OR ALL=(Enterococcus faecalis)) OR ALL=(Fusobacterium)) OR ALL=(Porphyromonas gingivalis)) OR ALL=(herpes virus)) OR ALL=(phyla)) OR ALL=(Taxa)) OR ALL=(molecular methods)) OR ALL=(molecular identification)) OR ALL=(Polymerase Chain Reaction)) OR ALL=(PCR)) OR ALL=(16 S rRNA)) OR ALL=(RNA, Ribosomal, 16 S)) OR ALL=(colony forming unit)) OR ALL=(CFU)) OR ALL=(taxonomy units)) OR ALL=(Mass Spectrometry)) OR ALL=(Mass Spectrum Analysis)) OR ALL=(Mass Spectrum Analyses)) OR ALL=(Mass Spectroscopy)) OR ALL=(Phylogeny)) OR ALL=(DNA sequence)) #1 AND #2 AND #3	2388
Google Scholar	(“microbiota” OR “bacterial species” OR “microbiome” OR “Polymerase chain reaction”) AND (“enterococcus faecalis” OR “fusobacterium” OR “porphyromonas gingivalis” OR “Herpes simplex virus” OR “phyla”) AND (“periapical lesion” OR “persistent endodontic infection” OR “secondary endodontic infection” OR “primary endodontic infection”) AND (“bone resorption” OR “apical periodontitis” OR “periapical radiograph” OR “CBCT”)	2890 (first 100 screened)

### Eligibility criteria

#### Inclusion criteria

- Study type: clinical trials, retrospective and observational studies.
- Studies done on systemically healthy patients.
- Studies done on permanent, mature teeth.
- Studies including patients with 1ry or 2ry (surgical or non-surgical) endodontic treatment.
- Patients with symptomatic or asymptomatic apical periodontitis.
- Studies reporting microbial and radiographic analysis.
- No time or age restrictions were applied.

#### Exclusion criteria

- Animal studies, case reports, case series.
- Reviews and surveys.
- Studies not in English language.
- No abstract available.
- Position statements and clinical guidelines.

### Data processing

The relevant studies that fulfilled the eligibility criteria were processed for data extraction into a standardized data extraction table by two reviewers (SN, ME), This was thereafter subjected to a countercheck by two more reviewers (MM, SS). For every paper, the following data were extracted: author(s) name, year of publication, country where the study was conducted, study design and study outcome measures (Table 2). Diagnosis of periapical pathosis, size of periapical lesion, radiographic analysis, microbial analysis and the most commonly found microorganisms (percentage of detection) (Table 3). Also, all microorganisms found in the included studies were listed (Table 4). The included publications were described using descriptive statistics, and the findings were explained through narrative synthesis. The methodological quality of the included studies was assessed using the JBI Critical Appraisal Tools (https://jbi.global/critical-appraisal-tools)specific to each study design.

**Table 2:** Main features of the included studies

Author/Year	Study design	Study location	Study outcomes measures
Amaral R R et al., 2022 [18]	randomized clinical trial	Brazil	analyze the endodontic microbial diversity
Orozco E et al., 2020 [20]	randomized clinical trial	Brazil	analyze the endodontic microbial diversity
Ping Y et al., 2015 [21]	randomized clinical trial	China	analyze the endodontic microbial diversity
Zahran S et al., 2022 [22]	randomized clinical trial	England	analyze endodontic microbial diversity after enhanced infection control protocol
Zahran S et al., 2021 [23]	randomized clinical trial	England	analyze endodontic microbial load after enhanced infection control protocol
Zandi H 2019 [24]	randomized clinical trial	Norway	analyze the endodontic microbial diversity
Sun X et al., 2022 [26]	observational clinical study	China	analyze the endodontic microbial diversity
Pérez-Carrasco V et al., 2023 [27]	observational clinical study	Spain	analyze the endodontic microbial diversity
Toia CC et al., 2022 [29]	randomized clinical trial	Brazil	analyze the endodontic microbial load and diversity
Arias-Moliz MT et al., 2024 [31]	observational clinical study	Spain	analyze the endodontic microbial diversity
Godoi-Jr EP et al., 2023 [32]	observational clinical study	Brazil	analyze the endodontic microbial diversity
Zargar N et al., 2019 [36]	observational clinical study	Iran	analyze the endodontic microbial diversity.
Zargar N et al., 2020 [44]	observational clinical study	Iran	analyze the endodontic microbial diversity
Didilescu AC et al., 2012 [50]	observational clinical study	Romania	analyze the presence of Parvimonas micra, Fusobacterium nucleatum, Campylobacter rectus, Eubacterium nodatum, Eikenella corrodens and apnocytophaga sputigena.
Makino K et al., 2015 [51]	observational clinical study	Japan	analyze the presence of EpsteinBarr virus (EBV) in endodontic infection
Ozbek SM et al., 2016 [52]	observational clinical study	Turkey	analyze the presence of Epstein–Barr virus (EBV), human cytomegalovirus (HCMV), human herpesvirus-6 (HHV-6) and human papillomaviruses (HPV) in endodontic infection
Siqueira JF Jr & ROCAS IN, 2002 [53]	observational clinical study	Brazil	analyze the presence of D pneumosintesin endodontic infection
Abushouk S et al., 2021 [54]	observational clinical study	Sudan	analyze the endodontic microbial diversity
Andric M et al., 2007 [55]	observational clinical study	Serbia	to analyze the presence of CMV in endodontic infection
Barani M et al., 2023 [56]	observational clinical study	Kosovo	To analyze the endodontic microbial diversity
Garcez AS et al., 2015 [57]	quasi-experimental studi	Brazil	analyze the microbial load in endodontic infection
Gomes BPFA et al., 2007 [58]	observational clinical study	Brazil	analyze the presence of Porphyromonas gingivalis, Treponema denticola, and Tannerella forsythia in endodontic infection
Sassone L et al., 2007 [59]	observational clinical study	Brazil	analyze the endodontic microbial diversity
Gomes BPFA et al., 2006 [60]	observational clinical study	Brazil	analyze the presence of Enterococcus faecalis in endodontic infection
Cao H et al., 2012 [61]	observational clinical study	China	analyze the prevalence of three black-pigmented bacterial species (Porphyromonas endodontalis, Porphyromonas gingivalis and Prevotella intermedia) in endodontic infection
Stojanović N et al., 2014 [62]	randomized clinical trial	Serbia	analyze the presence of Enterococcus faecalis and Porphyromonas gingivalis in endndontic infection
Gomes BPFA et al., 2005 [63]	observational clinical study	Brazil	analyze the presence of four black-pigmented bacteria, Porphyromonas gingivalis, Porphyromonas endodontalis, Prevotella intermedia and Prevotella nigrescens, in endodontic infections
Murad CF et al., 2014 [64]	observational clinical study	Brazil	analyze microbial diversity in endodontic infection
Foschi F et al., 2005 [65]	observational clinical study	Italy	analyze the presence of selected bacteria (Enterococcus faecalis, Porphyromonas gingivalis, Prevotella intermedia, Tannerella forsythensis, Treponema denticola) in endnontic infection
Hu Z et al., 2023 [66]	observational clinical study	China	analyze microbial diversity in endodontic infection
Gomes BP et al., 2021 [68]	observational clinical study	Brazil	analyze microbial diversity in endodontic infection
Moghadam MD et al., 2021 [70]	randomized clinical trial	Iran	analyze microbial load in endodontic infection
Nogales CG et al., 2025 [71]	quasi-experimental studi	Brazil	analyze microbial diversity in endodontic infection
Sedgley C et al., 2006 [72]	observational clinical study	USA	analyze presence of Enterococcus faecalis in endodontic infection
Siqueira JF Jr et al., 2016 [73]	observational clinical study	Brazil	analyze microbial diversity in endodontic infection
Gomes BPFA et al., 2015 [74]	observational clinical study	Brazil	analyze microbial diversity in endodontic infection
Zakaria MN et al., 2015 [75]	observational clinical study	Japan	analyze microbial diversity in endodontic infection
Sanghavi TH et al., 2014 [76]	observational clinical study	India	analyze the presence of Porphyromonas gingivalis, Treponema denticola, and Tannerella forsythia in endodontic infection
Blome B et al., 2008 [77]	observational clinical study	Germany	analyze microbial diversity in endodontic infection
Nobrega LM et al., 2016 [78]	observational clinical study	Brazil	analyze microbial diversity in endodontic infection
Arias-Moliz MT et al., 2024 [31]	observational clinical study	Spain	analyze microbial diversity in endodontic infection
Henriques LC et al., 2016 [79]	observational clinical study	Brazil	analyze microbial diversity in endodontic infection
Sunde PT et al., 2001 [80]	observational clinical study	Norway	analyze microbial diversity in endodontic infection
Ribeiro AC et al., 2011 [81]	observational clinical study	Brazil	analyze microbial diversity in endodontic infection
Jacinto RC et al., 2008 [82]	observational clinical study	Brazil	analyze presence of Fusobacterium nucleatum and Fusobacterium necrophorum in endodntic infection
Sassone LM et al., 2008 [83]	observational clinical study	Brazil	analyze microbial diversity in endodontic infection
Hou Y et al., 2021 [84]	observational clinical study	China	analyze microbial diversity in endodontic infection
Sabeti M and Slots J, 2004 [85]	observational clinical study	USA	analyze the of HCMV, EBV, and herpes simplex virus in endodontic infection
Geibel M et al., 2005 [86]	observational clinical study	Germany	analyze microbial diversity in endodontic infection
Cardoso FG et al., 2016 [87]	observational clinical study	Brazil	analyze microbial diversity in endodontic infection
Tiwari S et al., 2020 [88]	observational clinical study	India	analyze the presence of of Porphyromonas gingivalis, Treponema denticola, and Tannerella forsythia in endodontic infection
Bogen G and Slots J, 1999 [89]	observational clinical study	USA	analyze the presence of Porphyromonas endodontalis, Porphyromonas gingivalis, Prevotella intermedia and Prevotella nigrescens in endodontic infection
Machado et al., 2020 [93]	observational clinical study	Brazil	analyze microbial diversity and load in endodontic infection
Ahmed S et al. 2024 [94]	observational clinical study	Saudi Arabia	analyze the prevalence of E. faecalis and C. albicans in endodontic infection.
Kist S et al., 2017 [95]	randomized clinical trial	Germany	analyze microbial load in endodontic infection.
Donnermeyer D et al., 2025 [99]	observational clinical study	Germany	analyze microbial diversity in endodontic infection
Alquria TA et al., 2024 [102]	quasi-experimental studi	USA	analyze microbial diversity in endodontic infection
Pelozo LL et al., 2023 [106]	randomized clinical trial	Brazil	analyze microbial diversity and load in endodontic infection
Gomes BP et al., 2020 [107]	observational clinical study	Brazil	analyze the presence of F. alocis in endodontic infection.
Martinho FC et al., 2010 [109]	observational clinical study	Brazil	analyze microbial diversity in endodontic infection
Carvalho AP et al., 2020 [114]	observational clinical study	Brazil	analyze microbial load in endodontic infection
Cavalli D et al., 2017 [108]	randomized clinical trial	Brazil	analyze microbial diversity and load in endodontic infection
Card SJ et al., 2002 [115]	quasi-experimental studi	USA	analyze microbial load in endodontic infection
de Miranda RG and Colombo AP, 2018 [116]	randomized clinical trial	Brazil	analyze microbial load in endodontic infection
Buonavoglia A et al., 2023 [117]	observational clinical study	Italy	analyze microbial diversity in endodontic infection.
Bronzato JD et al., 2021 [118]	observational clinical study	Brazil	analyze microbial diversity in endodontic infection
Waltimo T et al., 2005 [119]	quasi-experimental studi	Finland	analyze microbial load in endodontic infection
Hepsenoglu YE and Ersahan S, 2023 [124]	randomized clinical trial	Turkey	analyze microbial load in endodontic infection
Ersahan S et al.,2022 [132]	randomized clinical trial	Turkey	analyze microbial diversity in endodontic infection
Brenda P. F. A et al., 2021 [68]	observational clinical study	Brazil	analyze microbial diversity in endodontic infection
Handal T et al., 2009 [19]	not mentioned	Norway	analyze the endodontic microbial diversity
Rodríguez R et al., 2023 [10]	not mentioned	Mexico	analyze the endodontic microbial diversity
Korona-Glowniak I et al., 2021 [25]	not mentioned	Poland	analyze the endodontic microbial diversity
Tennert C et al., 2014 [28]	not mentioned	Germany	analyze the endodontic microbial diversity
Shuping GB et al., 2000 [30]	not mentioned	USA	analyze the endodontic microbial load and diversity
Barbosa-Ribeiro M et al., 2020 [33]	not mentioned	Brazil	analyze the endodontic microbial diversity
Schirrmeister JF et al., 2009 [34]	not mentioned	Germany	analyze the endodontic microbial diversity
Schirrmeister JF et al., 2007 [35]	not mentioned	Germany	analyze the endodontic microbial diversity
Sánchez-Sanhueza G et al., 2018 [37]	not mentioned	Chile	analyze the endodontic microbial diversity
Siqueira JF et al., 2020 [38]	not mentioned	Brazil	analyze the endodontic microbial diversity
Cavrini F et al., 2008 [39]	not mentioned	Italy	analyze the presence of Treponema denticola in endodontic infection
Pirani C et al., 2008 [40]	not mentioned	Italy	analyse the presence of Enterococcus faecalis in endodontic infection
Teofani A et al., 2024 [41]	not mentioned	Italy	analyze the endodontic microbial diversity
Vianna ME et al., 2005 [42]	not mentioned	Brazil	analyze the endodontic microbial diversity
Siqueira Jr JF et al., 2000 [43]	not mentioned	Brazil	analyze the presence of Treponema denticola in endodontic infection
Lima AR et al., 2021 [45]	not mentioned	Brazil	analyze the presence of Streptococcus mutans in endodontic infection
Rosa TP et al., 2015 [46]	not mentioned	Brazil	analyze the presence of the Treponema species in endodontic infection
Qian W et al., 2019 [47]	not mentioned	China	analyze the endodontic microbial diversity
Assed S et al., 1996 [48]	not mentioned	Brazil	analyze anerobic microorganisms endodontic infection
Chávez De Paz LE et al., 2003 [49]	not mentioned	Sweden	analyze the endodontic microbial diversity
Zeledón R et al., 2015 [67]	not mentioned	Costa Rica.	analyze microbial diversity in endodontic infection
Peters LB and Wesselink PR, 2002 [69]	not mentioned	Netherlands	analyze microbial diversity in endodontic infection
Neves MA et al., 2020 [90]	not mentioned	Brazil	analyze microbial load in endodontic infection
Mussano F et al., 2018 [91]	not mentioned	Italy	analyze microbial diversity in endodontic infection
de Azevedo Moreira S 2021 [92]	not mentioned	Brazil	analyze the presence of Enterococcus faecalis and Actinomyces israelii in endodontic infection
Egan MW et al., 2002 [96]	not mentioned	England	analyze the diversity of yeasts in salivary and endodontic infection
Vianna ME et al., 2007 [97]	not mentioned	Brazil	analyze the presence of Synergistes phylotypes in endodontic infection
Siqueira JF and Rocas IN, 2003 [98]	not mentioned	Brazil	analyze the presence of Campylobacter gracilis and C. rectus in endodontic infection
Niazi SA et al., 2016 [100]	not mentioned	England	analyze the presence of y P. acnes in endodontic infection
Ahlat M et al., 2023 [101]	not mentioned	Turkey	analyze microbial diversity in endodontic infection
Blome B et al., 2008 [77]	not mentioned	Germany	analyze microbial diversity and load in endodontic infection
Hommez GM et al., 2004 [103]	not mentioned	Belgium	analyze microbial diversity in endodontic infection
Cardoso FG et al., 2015 [104]	not mentioned	Brazil	analyze microbial diversity in endodontic infection
Moraes SR et al., 2002 [105]	not mentioned	Brazil	analyze the clonal diversity of F. nucleatum strains in endodontic infection
Vianna ME et al., 2006 [6]	not mentioned	Brazil	analyze the presence of archaea in endodontic infections.
Carneiro E et al., 2017 [108]	not mentioned	Brazil	analyze the presence of gram-negative bacteria in endodontic infection
Garcez AS et al., 2008 [110]	not mentioned	Brazil	analyze microbial diversity and load in endodontic infection
Sabeti M et al., 2003 [111]	not mentioned	USA	analyze the presence of human cytomegalovirus (HCMV), Epstein-Barr virus (EBV) and herpes simplex virus (HSV) in endodontic infection
Tawfik SA et al., 2018 [112]	not mentioned	Egypt	analyze microbial diversity in endodontic infection
Siqueira Jr 2009 [9]	not mentioned	Brazil	analyze the presence of 81 species/phylotypes in abscess aspirates
Pereira R et al.,2017 [113]	not mentioned	Brazil	analyze microbial diversity in endodontic infection
Noguchi et al., 2005 [120]	not mentioned	Japan	analyze microbial diversity in extraradicular infection
Kesim B et al., 2023 [121]	not mentioned	Turkey	analyze microbial diversity in endodontic infection
Gajan EB et al., 2009 [122]	not mentioned	Iran	analyze microbial diversity in endodontic infection
Sabeti M et al., 2003 [123]	not mentioned	USA	analyze the presence of HCMV, EBV and herpes simplex virus (HSV) in endodontic infection
Sabeti M et al., 2003 [5]	not mentioned	USA	analyze the presence of HCMV and EBV and herpes simplex virus (HSV) in endodontic infection
Xavier A et al.,2014 [125]	not mentioned	Brazil	analyze the prevalence of E. faecalis in endodontic infection
Barbosa-ribeiro M et al., 2020 [33]	not mentioned	Brazil	analyze microbial diversity in endodontic infection
Abou-rass M and Bogen G,1998 [126]	not mentioned	USA	analyze microbial diversity in endodontic infection
Pinheiro E et al.,2002 [127]	not mentioned	Brazil	analyze microbial diversity in endodontic infection
Ozbek S et al.,2008 [128]	not mentioned	Turkey	analyze the presence of Enterococcus faecalis in endodontic infection
Martinho F et al.,2014 [129]	not mentioned	Brazil	analyze the presence of different Gram-negative bacterial species in endodontic infection
Pattanshetty S et al.,2017 [130]	not mentioned	India	analyze the presence of selective anaerobic microorganisms in endodontic infection
Sousa E et al.,2012 [131]	not mentioned	Brazil	analyze microbial diversity in endodontic infection
Al-Samahi S and A. Al-Omari M,2014 [133]	not mentioned	Saudi Arabia	analyze microbial diversity in endodontic infection
Heling I et al.,2001 [134]	not mentioned	Israel	analyze the presence of herpes simplex virus (HSV) in endodontic infection.
Tzanetakis G et al.,2015 [135]	not mentioned	Greece	analyze microbial diversity in endodontic infection
Kaufman B et al.,2005 [136]	not mentioned	USA	analyze the presence of E faecalis in endodontic infection
Tanumihardja M et al.,2014 [137]	not mentioned	Indonesia	analyze microbial diversity in endodontic infection
Zhang S et al., 2010 [138]	not mentioned	China	analyze microbial diversity in endodontic infection
Pinheiro E.T. et al.,2003 [127]	not mentioned	Brazil	analyze microbial diversity in endodontic infection
Jakovljevic et al., 2018 [160]	not mentioned	Serbia	analyze the relation between Epstein–Barr virus (EBV) and levels of oxidative stress biomarkers [8-hydroxydeoxyguanosine (8-OHdG) and oxidized glutathione (GSSG)] and bone resorption regulators [receptor activator of nuclear factor (NF-jB) ligand (RANKL) and osteoprotegerin (OPG)]

**Table 3:** Radiographic and microbial characteristics of the included studies

Author/Year	Size of periapical lesion	Radiographic analysis	Microbial detection method	Topmost commonly found microorganisms in infection site
Amaral R R et al., 2022 [18]	small (largest diameter < 5 mm) or large (largest diameter ≥ 5 mm).	periapical radiograph	molecular based	Most common microbiome: Firmicutes, Bacteroidetes, Proteobacteria, and Actinobacteria.
Handal T et al., 2009 [19]	small (largest diameter < 5 mm) or large (largest diameter ≥ 5 mm).	periapical radiograph	molecular based	Most common microbiome: Fusobacterium spp., Prevotella spp., Tannerella forsythia, Porphyromonas endodontalis, Treponema denticola, Bacteroidetes spp., Peptostreptococcus spp., and Streptococcus spp.
Rodríguez R et al., 2023 [10]	large (size from 5.60 mm3 to 50.31 mm3)	CBCT	Culture-Based	Most common microbiome: Actinomyces naeslundii, Enterococcus faecalis, Aerococcus viridans, Streptococcus sanguis, Fusobacterium nucleatum
Orozco E et al., 2020 [20]	large (size from 63.3 mm3 to 88.0 mm3)	CBCT	culture-based	Most common microbiome: S. constellatus, E. faecalis, F. nucleatum SP, P. gingivalis, P. melaninogenica and S. intermedius
Ping Y et al., 2015 [21]	small (largest diameter < 5 mm) or large (largest diameter ≥ 5 mm).	periapical radiograph	molecular based	Most common microbiome: seven phyla; Firmicutes, Proteobacteria, Bacteroidetes, Fusobacteria, Actinobacteria, Synergistetes, and Spirochaetes.
Zahran S et al., 2022 [22]	not mentioned	CBCT	molecular based	NA
Zahran S et al., 2021 [23]	not mentioned	CBCT	molecular based	NA
Zandi H 2019 [24]	small (largest diameter < 5 mm) or large (largest diameter ≥ 5 mm).	periapical radiograph	molecular based	NA
Korona-Glowniak I et al., 2021 [25]	not mentioned	periapical radiograph	culture and molecular based	Most common microbiomes: Firmicutes, Actinobacteria, Bacteroidetes, Proteobacteria and Fusobacteria. Enterococcus faecalis and Candida albicans detected in resistant infections.
Sun X et al., 2022 [26]	not mentioned	Periapical radiograph and CBCT	molecular based	Most common microbiomes: Proteobacteria, Firmicutes, Bacteroidetes, Actinobacteria At genus level: Fusobacteria, Fusobacterium, Morganella, Burkholderia, Porphyromonas, Streptococcus, and Bifidobacterium. In sinus tract: Porphyromonas, Eubacterium, Treponema, Phocaeicola
Pérez-Carrasco V et al., 2023 [27]	not mentioned	periapical radiograph	molecular based	Most common microbiome: phyla level Bacteroidetes, Fusobacteria Proteobacteria Synergistetes and Actinobacteria both in apices and periapical lesions. genera level; Fusobacterium Porphyromonas, Streptococcus, Pseudomonas, Fretibacterium and Tannerella, Phreatobacter, Afipia and Xanthobacteriaceae_
Tennert C et al., 2014 [28]	not mentioned	periapical radiograph	culture and molecular based	Most common microbiome: E. faecalis and Streptococcus mutans
Toia CC et al., 2022 [29]	not mentioned	CBCT	culture and molecular based	NA
Shuping GB et al., 2000 [30]	not mentioned	periapical radiograph	Culture Based	NA
Arias-Moliz MT et al., 2024 [31]	not mentioned	periapical radiograph	molecular based	Most common microbiome: Fusobacterium nucleatum, Prevotella loescheii Streptococcus intermedius, P. gingivalis, Parvimonas micra, Synergistetes bacterium, Tannerella forsythia, Peptostreptococcus stomatis, Pseudomonas gessardii and Pseudoramibacter alactolyticus
Godoi-Jr EP et al., 2023 [32]	small (largest diameter < 5 mm) or large (largest diameter ≥ 5 mm).	periapical radiograph	Culture and molecular based	Most common microbiome: Enterococcus faecalis, Fusobacterium nucleatum, Porphyromonas gingivalis, Parvimonas micra, and Aggregatibacter actinomycetemcomitans
Barbosa-Ribeiro M et al., 2020 [33]	not mentioned	periapical radiograph	molecular based	Most common microbiome: E. faecalis, Staphylococcus epidermidis, Streptococcus sanguis and A. viridans
Schirrmeister JF et al., 2009 [34]	not mentioned	periapical radiograph	molecular based	Most common microbiome: Solobacterium moorei and F. nucleatum.
Schirrmeister JF et al., 2007 [35]	not mentioned	periapical radiograph	molecular based	Most common microbiome: E faecalis
Zargar N et al., 2019 [36]	small (largest diameter < 5 mm) or large (largest diameter ≥ 5 mm).	periapical radiograph	culture and molecular based	Most common microbiome: Enterococcus faecalis, Prevotella pallens
Sánchez-Sanhueza G et al., 2018 [37]	not mentioned	periapical radiograph	molecular based	Most common microbiome: Proteobacteria, Bacteroidetes
Siqueira JF et al., 2020 [38]	small (largest diameter < 5 mm) or large (largest diameter ≥ 5 mm).	CBCT	molecular based	Most common microbiome: Actinobacteria counts, Streptococci, E. faecalis
Cavrini F et al., 2008 [39]	not mentioned	periapical radiograph	molecular based	Most common microbiome: T. denticola
Pirani C et al., 2008 [40]	small (largest diameter < 5 mm) or large (largest diameter ≥ 5 mm).	periapical radiograph	molecular based	Most common microbiome: E. faecalis
Teofani A et al., 2024 [41]	small (largest diameter < 5 mm) or large (largest diameter ≥ 5 mm).	periapical radiograph	molecular based	Most common microbiome: Firmicutes, Bacteroidetes, Proteobacteria, Fusobacteria, Synergistetes
Vianna ME et al., 2005 [42]	not mentioned	periapical radiograph	culture and molecular based	Most common microbiome found by culture: F. nucleatum, Gemella morbillorum, Eubacterium lentum, and Enterococcus faecalis. Most common microbiome found by the DNA chip: M. micros, F. nucleatum ssp., T. forsythia, and T. denticola.
Siqueira Jr JF et al., 2000 [43]	not mentioned	periapical radiograph	molecular based	Most common microbiome: T. denticola.
Zargar N et al., 2020 [44]	not mentioned	periapical radiograph	molecular based	Most common microbiome: Dialister invisus, Porphyromonas gingivalis, Streptococcus salivarius, Treponema denticola, Lysinibacillus fusiformis
Lima AR et al., 2021 [45]	not mentioned	periapical radiograph	molecular based	Most common microbiome: S. mutans
Rosa TP et al., 2015 [46]	not mentioned	periapical radiograph	culture and molecular based	Most common microbiome: Treponema species, T. socranskii, T. maltophilum, T. amylovorum, T. lecithinolyticum, T. denticola, T. pectinovorum, and T. medium
Qian W et al., 2019 [47]	small (largest diameter < 5 mm) or large (largest diameter ≥ 5 mm).	periapical radiograph	molecular based	Most common microbiota: Streptococcus, Haemophilus Actinomyces, Granulicatella, Leptotrichia.
Assed S et al., 1996 [48]	not mentioned	periapical radiograph	molecular based	Most common microbiota: Actinomyces viscosus (56%), Prevotella intermedia (48%), Fusobacterium nucleatum (40%), Porphyromonas gingivalis (16%).
Chávez De Paz LE et al., 2003 [49]	small (< 2 mm) or large (> 2 mm)	periapical radiograph	culture, Gas-liquid chromatography	Most common microbiome: Non-mutans group streptococci, Enterococcus spp., Coagulase negative staphylococci, Peptostreptococcus spp., Mutans group streptococci, Lactobacillus spp.
Didilescu AC et al., 2012 [50]	not mentioned	Periapical radiograph	molecular based	Most common microbiome: In endodontic samples: P. micra, F. nucleatum and C. sputigena. In periodontal samples: the same species, plus C. rectus.
Makino K et al., 2015 [51]	large (largest diameter ≥ 5 mm).	periapical radiograph	molecular based	Most common microbiome: EBV
Ozbek SM et al., 2016 [52]	small (largest diameter < 5 mm) or large (largest diameter ≥ 5 mm).	periapical radiograph	molecular based	Most common microbiome: HCMV, EBV, HPV and HHV-6
Siqueira JF Jr & ROCAS IN, 2002 [53]	not mentioned	periapical radiograph	molecular based	Most common microbiome: Dialister pneumosintes, D pneumosintes
Abushouk S et al., 2021 [54]	not mentioned	periapical radiograph	molecular based	Most common microbiome: F. nucleatum, T. denticola, P. endodontalis
Andric M et al., 2007 [55]	large (largest diameter ≥ 5 mm).	periapical radiograph	molecular based	Most common microbiome: Cytomegalo Virus (CMV)
Barani M et al., 2023 [56]	not mentioned	Periapical radiograph	culture based	Most common microbiome: Streptococcus mitis, Streptococcus oralis, Granulicatella elegans, Enterococcus faecalis, Clostridium bifermentans
Garcez AS et al., 2015 [57]	not mentioned	periapical radiograph	Culture Based Methods.	NA
Gomes BPFA et al., 2007 [58]	not mentioned	periapical radiograph	molecular based	Most common microbiome: Porphyromonas gingivalis Treponema denticola, Tannerella forsythia
Sassone L et al., 2007 [59]	not mentioned	periapical radiograph	molecular based	Most common microbiome: Enterococcus faecalis, Campylobacter gracilis, Leptotrichia buccalis, Neisseria mucosa, Prevotella melaninogenica, Fusobacterium nucleatum ssp. vincentii
Gomes BPFA et al., 2006 [60]	not mentioned	periapical radiograph	culture and molecular based	Most common microbiome: Enterococcus faecalis
Cao H et al., 2012 [61]	not mentioned	periapical radiograph	molecular based	Most common microbiome: Porphyromonas endodontalis, Prevotella intermedia, Porphyromonas gingivalis
Stojanović N et al., 2014 [62]	not mentioned	periapical radiograph	molecular based	Most common microbiome: Enterococcus faecalis, Porphyromonas gingivalis
Gomes BPFA et al., 2005 [63]	not mentioned	periapical radiograph	culture and molecular based	Most common microbiome: by molecular analysis: Porphyromonas gingivalis, Prevotella intermedia, Porphyromonas endodontalis, Prevotella nigrescens.by culture analysis: Prevotella corporis, P. loescheii, P. denticola, P. melaninogenica
Murad CF et al., 2014 [64]	small or large (range from 0.82 mm to 50.95 mm)	periapical radiograph	molecular based	Most common microbiome: Enterococcus faecium Staphylococcus epidermidis, Eubacterium saburreum, Parvimonas micra Streptococcus sanguis, Capnocytophaga sputigena, Leptotrichia buccalis, Enterococcus faecalis, Staphylococcus warneri
Foschi F et al., 2005 [65]	small (largest diameter < 5 mm) or large (largest diameter ≥ 5 mm).	periapical radiograph	molecular based	Most common micrbiome: T. denticola and E. faecalis, P. gingivalis, P. intermedia, and T. forsythensis
Hu Z et al., 2023 [66]	small (largest diameter < 5 mm)	periapical radiograph	molecular based	Most common microbiome: Porphyromonas gingivalis, Fusobacterium nucleatum, Pyramidobacter piscolens, Dialister invisus, Porphyromonas endodontalis, Parvimonas micra, Filifactor alocis, Prevotella oris, Phocaeicola abscessus
Zeledón R et al., 2015 [67]	large (largest diameter ≥ 5 mm).	periapical radiograph	molecular based	Most common microbiome: Actinomyces Israelii, Enterococcus Faecalis, Fusobacterium Nucleatum/Prevotella Nigrescens, Phorphyromonas Endodontalis.
Gomes BP et al., 2021 [68]	small (largest diameter < 5 mm) or large (largest diameter ≥ 5 mm).	periapical radiograph	Culture and molecular based	Most common microbiome detected by molecular method: molecular analyses; P. gingivalis, E. faecalis, and Fusobacterium nucleatum. using culture method : E. faecalis, G. morbillorum, Aerococcus viridans, Gemella haemolysans, and Staphylococcus lentus
Peters LB and Wesselink PR, 2002 [69]	small (largest diameter 0.5 mm).	periapical radiograph and CBCT	Culture Based	NA
Moghadam MD et al., 2021 [70]	small ≤ 5 mm or large > 5	CBCT	culture based	NA
Nogales CG et al., 2025 [71]	not mentioned	periapical radiograph	molecular based	NA
Sedgley C et al., 2006 [72]	not mentioned	periapical radiograph	Culture and molecular based	Most common microbiome: Using molecular analysis E. faecalis culture analysis: Streptococcus sanguis, Proteus spp., non-polysaccharide producing Streptococcus spp., and gram-positive anaerobic rods.
Siqueira JF Jr et al., 2016 [73]	not mentioned	Periapical radiograph and CBCT	molecular based	Most common microbiomes: Phyla level; Proteobacteria, Firmicutes, Fusobacteria, Actinobacteria. Genera level; Fusobacterium, Pseudomonas.
Gomes BPFA et al., 2015 [74]	not mentioned	periapical radiograph	culture and molecular based	Most common microbiomes: Enterococcus faecalis, Parvimonas micra, Mogibacterium timidum, Filifactor alocis, and Fretibacterium fastidiosum.
Zakaria MN et al., 2015 [75]	small (largest diameter < 5 mm) or large (largest diameter ≥ 5 mm).	periapical radiograph	molecular based	Most common microbiome: Porphyromonas gingivalis, Propionibacterium acnes, Fusobacterium nucleatum, Streptococcus mitis, Peptostreptococcaceae sp.
Sanghavi TH et al., 2014 [76]	not mentioned	periapical radiograph	molecular based	Most common microbiome: Treponema denticola, Porphyromonas gingivalis, Tannerella forsythia
Blome B et al., 2008 [77]	not mentioned	periapical radiograph	molecular based	Most common microbiome: Peptostreptococcus micros, P. endodontalis, T. denticola, T. forsythia, F. nucleatum
Nobrega LM et al., 2016 [78]	not mentioned	periapical radiograph	molecular based	Most common microbiome: Prevotella spp. (e.g., Prevotella oris), Fusobacterium nucleatum, Filifactor alocis, Peptostreptococcus stomatis, Dialister invisus, Phocaeicola abscessus
Arias-Moliz MT et al., 2024 [31]	not mentioned	periapical radiograph	molecular based	Most common microbiome: Fusobacterium nucleatum, Prevotella loescheii, Streptococcus intermedius, Porphyromonas gingivalis Parvimonas micra, Synergistetes bacterium
Henriques LC et al., 2016 [79]	not mentioned	periapical radiograph	molecular based	Most common microbiome: Corynebacterium diphtheriae, Porphyromonas gingivalis, Streptococcus sobrinus, Stenotrophomonas maltophilia
Sunde PT et al., 2001 [80]	not mentioned	periapical radiograph	molecular based	Most common microbiome: Streptococcus gordonii, Selenomonas noxia, Prevotella intermedia, Capnocytophaga gingivalis, Fusobacterium nucleatum ssp. polymorphum, Treponema denticola, Fusobacterium nucleatum ssp. vincentii, Veillonella parvula, Streptococcus anginosus, Streptococcus gordonii, Peptostreptococcus micros, Actinomyces israelii
Ribeiro AC et al., 2011 [81]	not mentioned	periapical radiograph	molecular based	Most common microbiome: Atopobium rimae, Dialister invisus, Prevotella oris, Pseudoramibacter alactolyticus, Tannerella forsythia
Jacinto RC et al., 2008 [82]	not mentioned	periapical radiograph	Culture based	Most common microbiome: Fusobacterium nucleatum, Fusobacterium necrophorum
Sassone LM et al., 2008 [83]	not mentioned	periapical radiograph	molecular based	Most common microbiome: Fusobacterium nucleatum ssp. vincentii, Veillonella parvula, Treponema socranskii, Enterococcus faecalis, Campylobacter gracilis.
Hou Y et al., 2021 [84]	not mentioned	periapical radiograph	molecular based	Most common microbiome: at Genera level; Porphyromonas, Fusobacterium, Faecalibacterium, Prevotella, Phocaeicola, Streptococcus, Parvimonas.
Sabeti M and Slots J, 2004 [85]	not mentioned	periapical radiograph	Culture based, viral detection	Most common microbiome: Streptococcus, Fusobacterium, P. micros, Staphylococcus, Campylobacter
Geibel M et al., 2005 [86]	not mentioned	periapical radiograph	molecular based	Most common microbiome: Micromonas micros (gram positive coccus), F. nucleatum, E. faecalis, S. sanguinis, E. coli or P. aeruginosa
Cardoso FG et al., 2016 [87]	not mentioned	periapical radiograph	Culture; and PCR	Most common microbiome: Porphyromonas endodontalis
Tiwari S et al., 2020 [88]	not mentioned	periapical radiograph	molecular based	Most common microbiome: P. gingivalis most prevalent, followed by T. forsythia and T. denticola
Bogen G and Slots J, 1999 [89]	not mentioned	periapical radiograph	Culture and molecular based	NA
Neves MA et al., 2020 [90]	not mentioned	periapical radiograph	molecular based	NA
Mussano F et al., 2018 [91]	large (< 10 mm)	panoramic radiograph	molecular based	Most common microbiome: Lactococcus lactis, Propionibacterium acnes, Staphylococcus warneri, Acinetobacter johnsonii and Gemellales. L. lactis
de Azevedo Moreira S 2021 [92]	not mentioned	periapical radiograph	molecular based	Most common microbiome: Enterococcus faecalis
Machado et al., 2020 [93]	not mentioned	periapical radiograph and CBCT.	culture technique and molecular based	Most common microbiome: gram-positive bacterial species (S. intermedius, E. faecalis, P. acnes, and S. constellatus. gram-negative bacterial species P. gingivalis, C. sputigena, P. melaninogenica, and L. buccalis
Ahmed S et al. 2024 [94]	not mentioned	periapical radiograph	Culture-Based Methods.	Most common microbiome: E. faecalis and C. albicans
Kist S et al., 2017 [95]	not mentioned	periapical radiograph	molecular based	Most common microbiome: facultative anaerobe Gram-positive cocci Streptococcus spp, Gram-positive anaerobe cocci Parvimonas spp, and obligate anaerobic Gram-negative rods Prevotella spp.
Egan MW et al., 2002 [96]	not mentioned	periapical radiograph	Molecular Based	Most common microbiome: Candida albicans and Rodotorula mucilaginosa
Vianna ME et al., 2007 [97]	not mentioned	periapical radiograph	molecular based	Most common microbiome: Treponema, Porphyromonas gingivalis, Prevotella intermedia
Siqueira JF and Rocas IN, 2003 [98]	not mentioned	periapical radiograph	molecular based	Most common microbiome: Campylobacter gracilis and C. rectus
Donnermeyer D et al., 2025 [99]	not mentioned	Periapical radiograph	molecular based	Most common microbiome: Pseudomonadales,
Niazi SA et al., 2016 [100]	not mentioned	Periapical radiograph	molecular based	Most common microbiome: Propionibacterium acnes Actinomyces naeslundii, Streptococcus gordonii, Streptococcus mitis bv, Streptococcus sanguinis, V. dispar and V. parvula Propionibacterium acnes, Staphylococcus epidermidis
Ahlat M et al., 2023 [101]	not mentioned	Periapical radiograph	molecular based	Most common microbiome: Bacillota, Bacteroidota, Pseudomonadota, Fusobacteriota, Ascomycota, Enterococcus faecalis, Lactobacillus rhamnosus’’
Alquria TA et al., 2024 [102]	not mentioned	Periapical radiograph	molecular based	Most common microbiomes: Bacteroidetes, Firmicutes, and Synergistetes
Blome B et al., 2008 [77]	not mentioned	Periapical radiograph	molecular based	Most common microbiome: P. micros and P. endodontalis
Hommez GM et al., 2004 [103]	not mentioned	periapical radiograph	molecular based	Most common microbiome: Fusobacterium nucleatum/Streptococcus mitis, Veillonella sp.
Cardoso FG et al., 2015 [104]	not mentioned	CBCT	Culture Based Method.	NA
Moraes SR et al., 2002 [105]	not mentioned	periapical radiograph	culture and molecular based	NA
Pelozo LL et al., 2023 [106]	not mentioned	periapical radiograph	Culture Based Method	NA
Vianna ME et al., 2006 [6]	not mentioned	periapical radiograph	molecular based	Most common microbiome: Methanobrevibacter oralis-like phylotype
Gomes BP et al., 2020 [107]	not mentioned	periapical radiograph	molecular based.	Most common microbiome: F. alocis
Carneiro E et al., 2017 [139]	not mentioned	Periapical radiograph	Immunohistochemistry and gram stain	NA
Martinho FC et al., 2010 [109]	small (< 2 mm) or large (≥ 2 mm)	periapical radiograph	molecular based	Most common microbiome: Prevotella nigrescens
Garcez AS et al., 2008 [110]	not mentioned	periapical radiograph	culture based	NA
Sabeti M et al., 2003 [123]	large (largest diameter ≥ 15 mm)	periapical radiograph	molecular based	Most common microbiome: HCMV and EBV, HCMV
Tawfik SA et al., 2018 [112]	not mentioned	Periapical radiograph	molecular based	Most common microbiome: At phyla level; Firmicutes, Bacteroidetes, Proteobacteria, and Synergistetes. At genus level; Prevotella, Bacillus, Porphyromonas, Streptococcus, and Bacteroides.
Siqueira Jr 2009 [9, 140]	not mentioned	Periapical radiograph	molecular based	Most common microbiome: Fusobacterium nucleatum, Parvimonas micra, Porphyromonas endodontalis, Olsenella uli, streptococci, Eikenella corrodens,
Pereira R et al.,2017 [113]	not mentioned	Periapical radiograph	molecular based	Most common microbiome: Fusobacterium nucleatum, Dialister pneumosintes and Tannerella forsythia, Dialister pneumosintes
Carvalho AP et al., 2020 [114]	not mentioned	Periapical radiograph	molecular based	NA
Cavalli D et al., 2017 [108]	not mentioned	Periapical radiograph	culture and molecular based	NA
Card SJ et al., 2002 [115]	not mentioned	Periapical radiograph	Culture method and PMR method	NA
de Miranda RG and Colombo AP, 2018 [116]	not mentioned	Periapical radiograph	molecular based	Most common microbiome: Candida albicans, Dialister pneumosintes, Prevotella nigrescens, Prevotella tannerae, and Peptostreptococcus anaerobius
Buonavoglia A et al., 2023 [117]	not mentioned	periapical radiograph	molecular based	Most common microbiome: at the phylum level Firmicutes, Bacteroidetes, Actinobacteria.at genera level: Phocaeicola, Pseudomonas.Rothia, and Prevotella
Bronzato JD et al., 2021 [118]	not mentioned	Periapical radiograph and CBCT images	molecular based	Most common microbiome: Parvimonas micra, Enterococcus faecalis, Fusobacterium nucleatum and Porphyromonas endodontalis.
Waltimo T et al., 2005 [119]	not mentioned	Periapical radiograph	culture and molecular based	NA
Noguchi et al., 2005 [120]	not mentioned	periapical radiograph	molecular based	Most common microbiome: Fusobacterium nucleatum, Porphyromo nas gingivalis, and Tannellera forsythensis
Kesim B et al., 2023 [121]	large (largest diameter > 2.5 mm)	periapical radiograph	molecular based	Most common microbiome: Gram-negative facultative anaerobic Gammaproteobacteria class outgroup, two orders (Pasteurellales, Vibrionales), two families (Pasteurellaceae, Vibrionaceae), Gram-positive bacteria, Actinomycetales order, and Gram-positive anaerobic taxa, one genus (Olsenella) and one species (Olsenella uli),
Gajan EB et al., 2009 [122]	not mentioned	periapical radiograph	Culture Based Method.	Most common microbiome: Peptostreptococcus, Streptococcus, Porphyromonas, and Enterococcus faecalis. Strains of P. provetti, S. sanguis, S. salivarius, P. endodontalis, E. faecalis, C. albicans, Veillonella spp., E. coli, Actinomyces meteri, Fusobacterium, Eubacterium lertum, and S. oralis S. salivarius, P. endodontalis, A. odontolyticus, and Peptostreptococcus provetti
Sabeti M et al., 2003 [123]	large (largest diameter ≥ 15 mm)	periapical radiograph	molecular based.	Most common microbiome: HCMV and EBV
Sabeti M et al., 2003 [5]	large (largest diameter ≥ 15 mm)	periapical radiograph	molecular based	Most common microbiome: HCMV and EBV
Hepsenoglu YE and Ersahan S, 2023 [124]	not mentioned	periapical radiograph	molecular based	Most common microbiomes: Enterococcus, Streptococcus, Lactobacillus, Fusobacterium i, Bacillus, Neisseria, Alkalibacterium and Pseudoramibacter.
Xavier A et al.,2014 [125]	large (largest diameter ≥ 3 mm)	periapical radiograph	culture and molecular based	Most common microbiome: Enterococcus faecalis strains
Barbosa-Ribeiro M et al., 2020 [33]	large (largest diameter ≥ 3 mm)	Periapical radiograph	culture and molecular based	Most common microbiome: E. faecalis, P. gingivalis, F. nucleatum and A. actinomycetemcomitans.
Abou-Rass M and Bogen G,1998 [126]	not mentioned	Periapical radiographs	culture based	Most common microbiome: Actinomyces sp., Propionibacterium sp., Streptococcus sp, Staphlyococcus sp., Porphyromonas gingivalis, Peptostreptococcus micros and Gram-negative enterics.
Pinheiro E et al.,2002 [141]	not mentioned	Periapical radiographs	Culture method (advanced techniques for anaerobic species)	Most common microbiome: Facultative anaerobic species, Gram-positive microorganisms.
Ozbek S et al.,2008 [128]	not mentioned	periapical radiographs	culture method and Real-time PCR SYBR Green	Most common microbiome: E. faecalis
Martinho F et al.,2014 [129]	small (< 2 mm) or large (≥ 2 mm)	periapical radiographs	molecular based	Most common micrbiome: Prevotella nigrescens.
Pattanshetty S et al.,2017 [130]	not mentioned	periapical radiographs	molecular based	Most common microbiome: Treponema denticola (T. denticola)
Sousa E et al.,2012 [131]	not mentioned	periapical radiographs	culture based	Most common microbiome: A. prevotii, P. micra, and F. necrophorum.
Ersahan S et al.,2022 [132]	small (< 2 mm) or large (≥ 2 mm)	periapical radiographs	molecular based	Most common microbiome: Enterococcus, Streptococcus, Enterobacter, Eubacterium, Lactobacillus and Bacillus.
Al-Samahi S and A. Al-Omari M,2014 [133]	not mentioned	periapical radiographs	molecular based	Most common microbiome: Actinobacteria, Firmicute, Proteobacteria and Bacteroides
Heling I et al.,2001 [134]	not mentioned	periapical radiographs	molecular based	NA
Tzanetakis G et al.,2015 [135]	not mentioned	periapical radiographs	molecular based	Most common microbiome: Bacteroidetes, Proteobacteria and Tenericutes.at genus level: Lactobacillus, Streptococcus, and Sphingomonas
Kaufman B et al.,2005 [136]	not mentioned	periapical radiographs	molecular based	Most common microbiome: Enterococcus spp.
Tanumihardja M et al.,2014 [137]	not mentioned	periapical radiographs	Cultura based	Most Common microbiome: Porphyromonas spp, Streptococcus spp and Porphyromonasspp
Zhang S et al., 2010 [138]	not mentioned	periapical radiographs	molecular based	Most common microbiome: Porphyromonas endodontalis, Actinomyces viscosus, Candida albicans and Porphyromonas gingivalis. Fusobacterium, Actinomyces israelii and Enterococcus faecalis
Brenda P. F. A et al., 2021 [68]	not mentioned	periapical radiographs	culture and molecular based	Most common microbiom: by culture analysis Enterococcus faecalis and Gemella morbillorum. Molecular analyses : P. gingivalis, E. faecalis, and Fusobacterium nucleatum.
Pinheiro E.T. et al.,2003 [127]	not mentioned	periapical radiographs	Cultura based	Most common microbiom: Enterococcus, Streptococcus Peptostreptococcus, Actinomyces, Prevotella, Staphylococcus, Gemella, Fusobacterium, Veillonella, Lactobacillus, Propionibacterium and Haemophilus
Jakovljevic et al. 2018 [160]	Small (> 2 mm), large (> 12 mm)	periapical radiograph	molecular based	Most common microbiome : EBV

**Table 4:** Types of bacteria among studies with large and small lesions

Large lesions(types)	percentage	Frequency	Small lesions(types)	percentage	frequency
Enterococcus faecalis	45.5	15	Enterococcus faecalis	43.5	10
Streptococcus spp.(sanguis, intermedius, constellatus, mitis	33.3	11	Streptococcus spp.(sanguis, intermedius, constellatus, mitis	39.1	9
Fusobacterium(nucleatum	30.3	10	Fusobacterium (nucleatum	30.4	7
Prevotella	18.2	6	Prevotella	21.7	5
Porphyromonas gingivalis	18.2	6	Porphyromonus gingivalis	21.7	5
Treponema	15.2	5	Bacteroidetes	13	3
Actinomyces naeslundi	15.2	5	Actinomyces naeslundi	13	3
HCMV	15.2	5	Leptotrichia	13	3
Bacteroidetes	12.1	4	Staphylococci	13	3
Actinobacteria	12.1	4	Firmicutes	8.7	2
Staphylococcus	12.1	4	Actinobacteria	8.7	2
EBV	12.1	4	porphyromonus genera	8.7	2
Proteobacteria	9.1	3	Treponema	8.7	2
Peptostreptococcus	9.1	3	Tannerella forsythia	8.7	2
Porphyromonas endodontalis	9.1	3	Peptostreptococcus	8.7	2
Leptotrichia	9.1	3	Porphyromonas endodontalis	8.7	2
porphyromonus	6.1	2	Synergistetes	8.7	2
Acinetobacter	6.1	2	Capnocytophaga	8.7	2
Tannerella forsythia	6.1	2	Parvimonas micra	8.7	2
Aerococcus viridans	6.1	2	Dialister invisus	8.7	2
Synergistetes	6.1	2	Lactobacillus spp.	8.7	2
Capnocytophaga	6.1	2	Proteobacteria	4.3	1
Parvimonas micra	6.1	2	Pseudoramibacter	4.3	1
Aggregatibacter actinomycetemcomitans	6.1	2	Oribacterium	4.3	1
Lactobacillus spp.	6.1	2	Treponema denticola	4.3	1
HPV	6.1	2	Aerococcus viridans	4.3	1
Gemella	6.1	2	Spirochaetes	4.3	1
Firmicutes	3	1	Burkholderia	4.3	1
Pseudoramibacter	3	1	Veillonella	4.3	1
Oribacterium	3	1	Aggregatibacter actinomycetemcomitans	4.3	1
Treponema denticola	3	1	Granulicatella	4.3	1
Spirochaetes	3	1	EBV	4.3	1
Burkholderia	3	1	HCMV	4.3	1
Veillonella	3	1	Enterococcus faecium	4.3	1
Dialister invisus	3	1	Eubacterium saburreum	4.3	1
Granulicatella	3	1	Gemella	4.3	1
HHV-6	3	1	Propionibacterium acnes	4.3	1
Enterococcus faecium	3	1	Bacillus	4.3	1
Eubacterium saburreum	3	1	Enterobacter	4.3	1
Propionibacterium acnes	3	1	candida albicans	4.3	1
Lactococcus lactis	3	1	HSV1	4.3	1
Olsenella	3	1	Pyramidobacter piscolens	4.3	1
Bacillus	3	1	Filifactor alocis	4.3	1
Enterobacter	3	1	Phocaeicola abscessus	4.3	1
Haemophilus	0	0			

Numerical data were summarized using means and SD, whereas categorical data were described using frequencies and percentages. The statistical analysis was carried out by utilizing IBM, SPSS version 23 for Windows, Armonk, NY, USA.

## Results

**Fig. 1:**
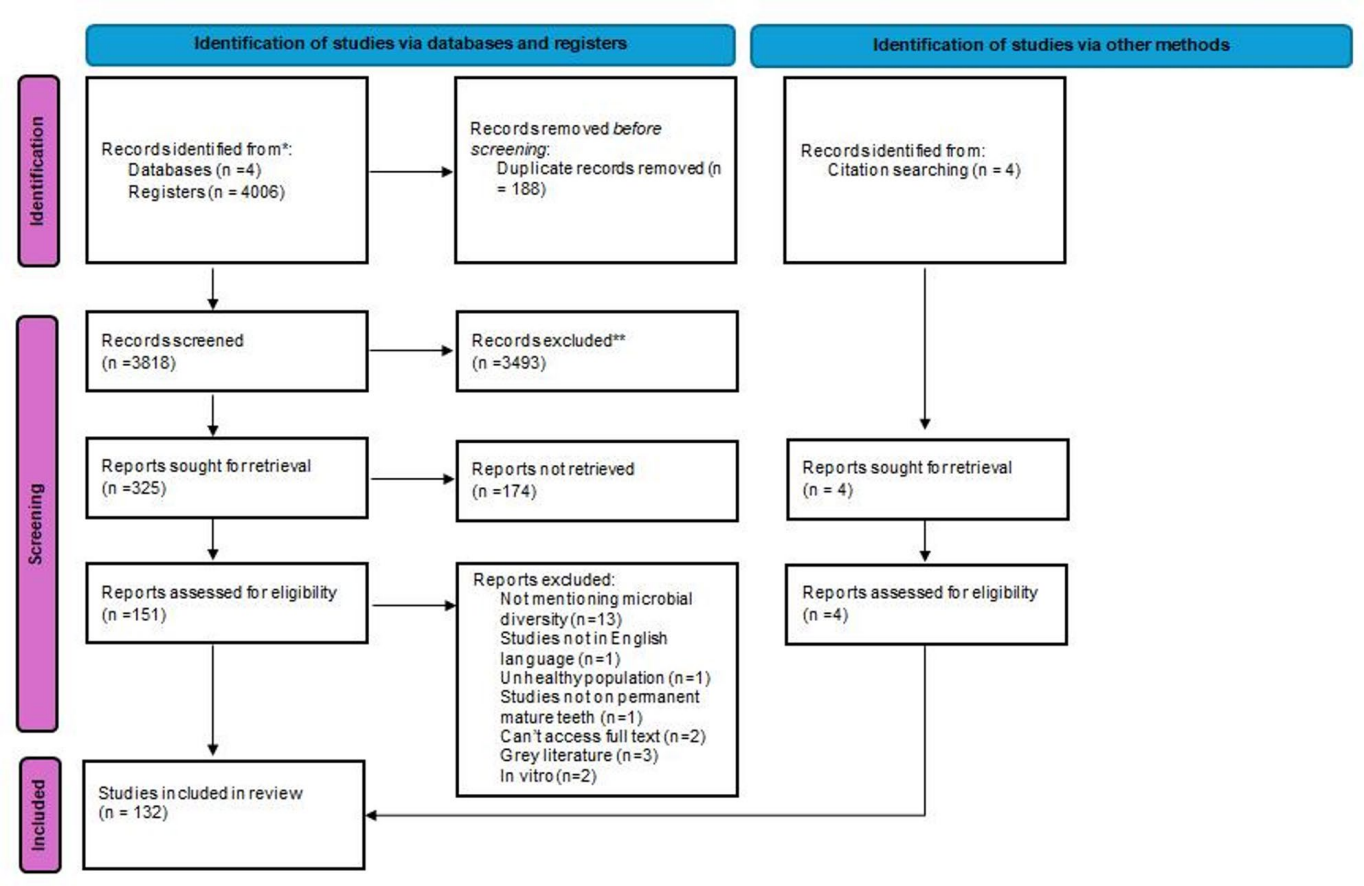
The review process Prisma flow chart [17]

The search identified 4006 potentially relevant records retrieved from online databases search (PubMed = 1038, Scopus = 480, Web of Science = 2388, Google scholar = 100 (top 100 relevant studies) in addition to four records retrieved via handsearching. After duplicates removal 3818 records were retained for screening. An initial screening resulted in exclusion of 3493 records based on titles and abstracts. A further 174 records were excluded following the full text screening. Eventually, 132 studies met the inclusion criteria and were processed for data extraction and synthesis Fig. 1 [17].

### General characteristics of the included studies

Table 2 summarizes the general characteristics of the included studies. Randomized clinical trials accounted for 11.36% (*n* = 15) of the studies, while quasi-experimental designs were identified in 3.79% (*n* = 5). Observational clinical studies comprised 37.88% (*n* = 50) of the included articles. Notably, 46.97% of the studies did not explicitly report the clinical study design.

### Quality assessment and risk of bias

Risk of bias was assessed using JBI Checklist for Randomized Controlled Trials (13 items), Checklist for Quasi-Experimental Studies (9 items) and Checklist for Analytical Cross-Sectional Studies (8 items). [https://jbi.global/critical-appraisal-tools] All randomized controlled trials were judged to have a moderate risk of bias, mainly due to unclear allocation concealment and lack of blinding, despite generally appropriate randomization procedures, outcome measurement, and statistical analysis (Supplementary Table 1). All quasi-experimental studies were assessed as having a moderate risk of bias, mainly due to the absence of appropriate control groups despite otherwise adequate methodological conduct (Supplementary Table 2). Most observational studies demonstrated acceptable methodological quality; however, inadequate identification and management of confounding factors resulted in a moderate risk of bias in a substantial proportion of the included evidence (Supplementary Table 3).

### Outcome measures

Regarding outcome measures, most of the studies assessed microbial diversity (58%), followed by (34.4%) that evaluated selected pathogens and only (9.2%) of studies measured the microbial load. (supplementary Table 5).

**Fig. 2:**
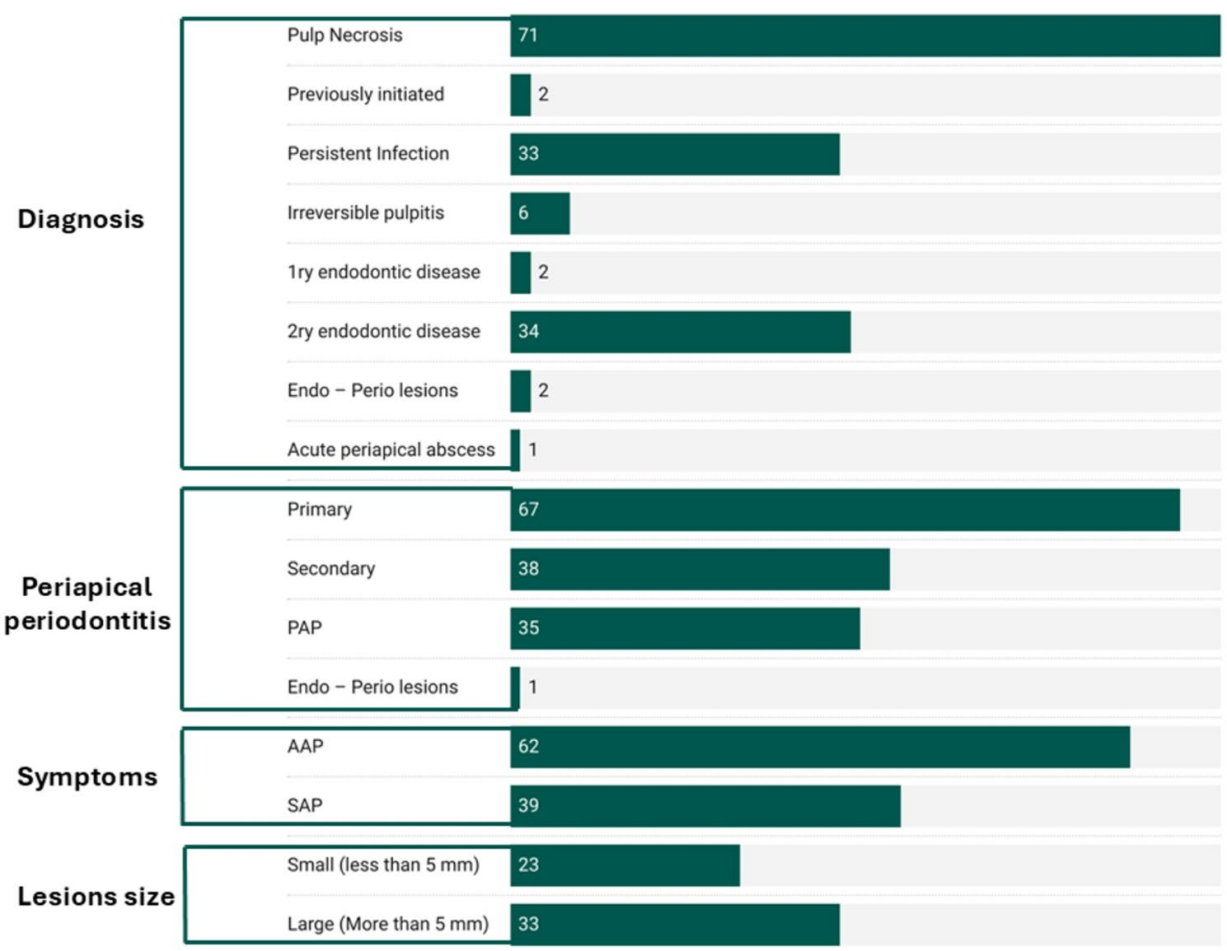
Study characteristics of the included studies

Figure 2 shows the different pulpal, apical periodontitis diagnosis, associated symptoms and apical lesion size.

**Fig. 3:**
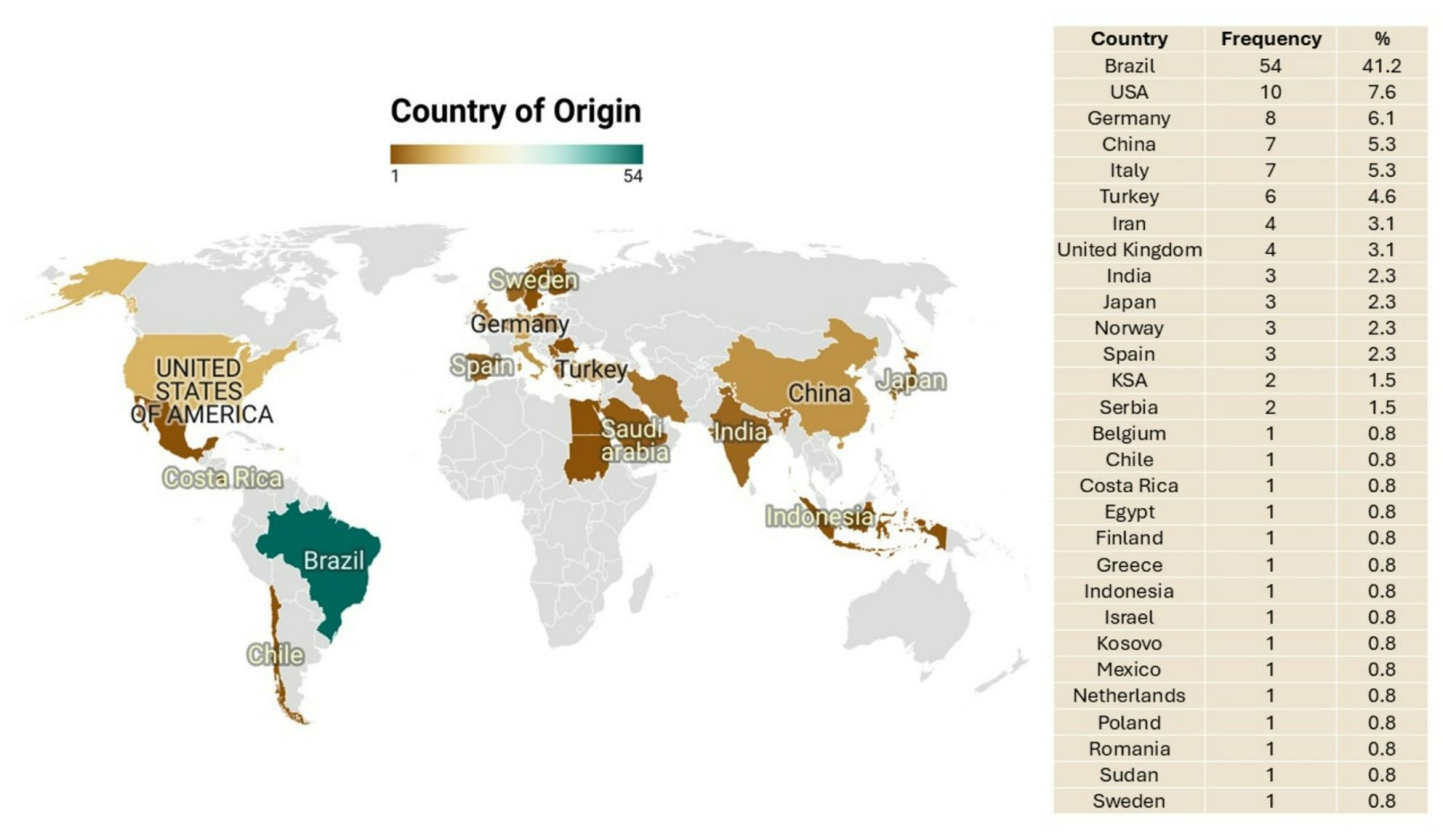
Map of the studies included in the scoping review

**Fig. 4:**
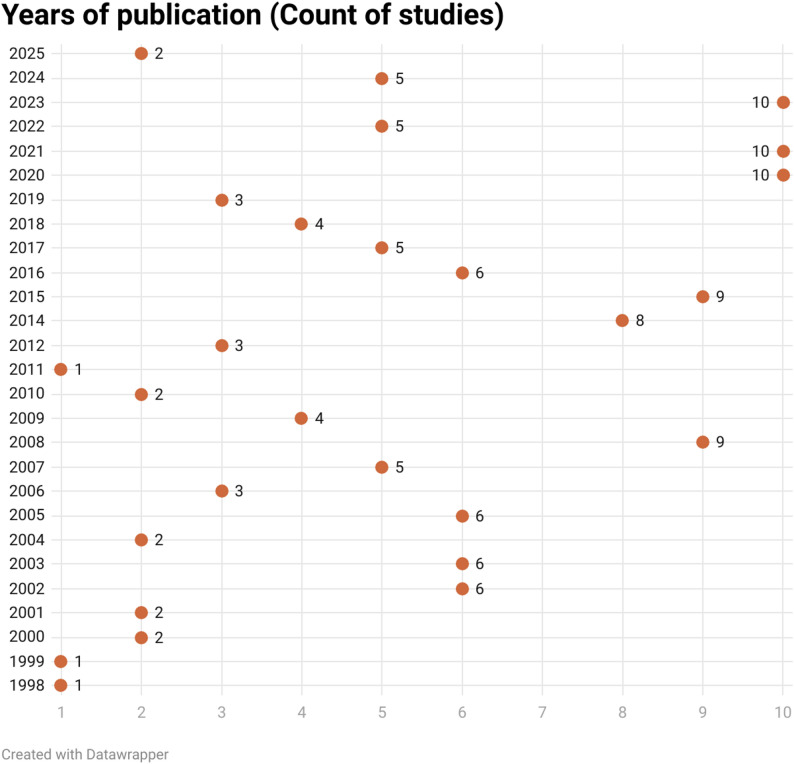
Year of publications (counts of studies)

Global distribution of the included studies (Fig. 3) showed Brazil, USA, Germany, China and Italy were found to have the most published articles among publishing nations (54, 10, 8, 7 and 7 studies, respectively).Time distribution of the included studies (Fig. 4) showed consistency over the years in the field of research on bacterial diversity and microbial load in relation to apical infections.

### Outcome characteristics of the included studies and detection methods (Fig. 5)

The majority of the studies (104/131) employed non-surgical sampling (using paper points or hand files from the main root canal) and only 29/131 studies had taken the samples surgically (root apices or apical lesion).

**Fig. 5:**
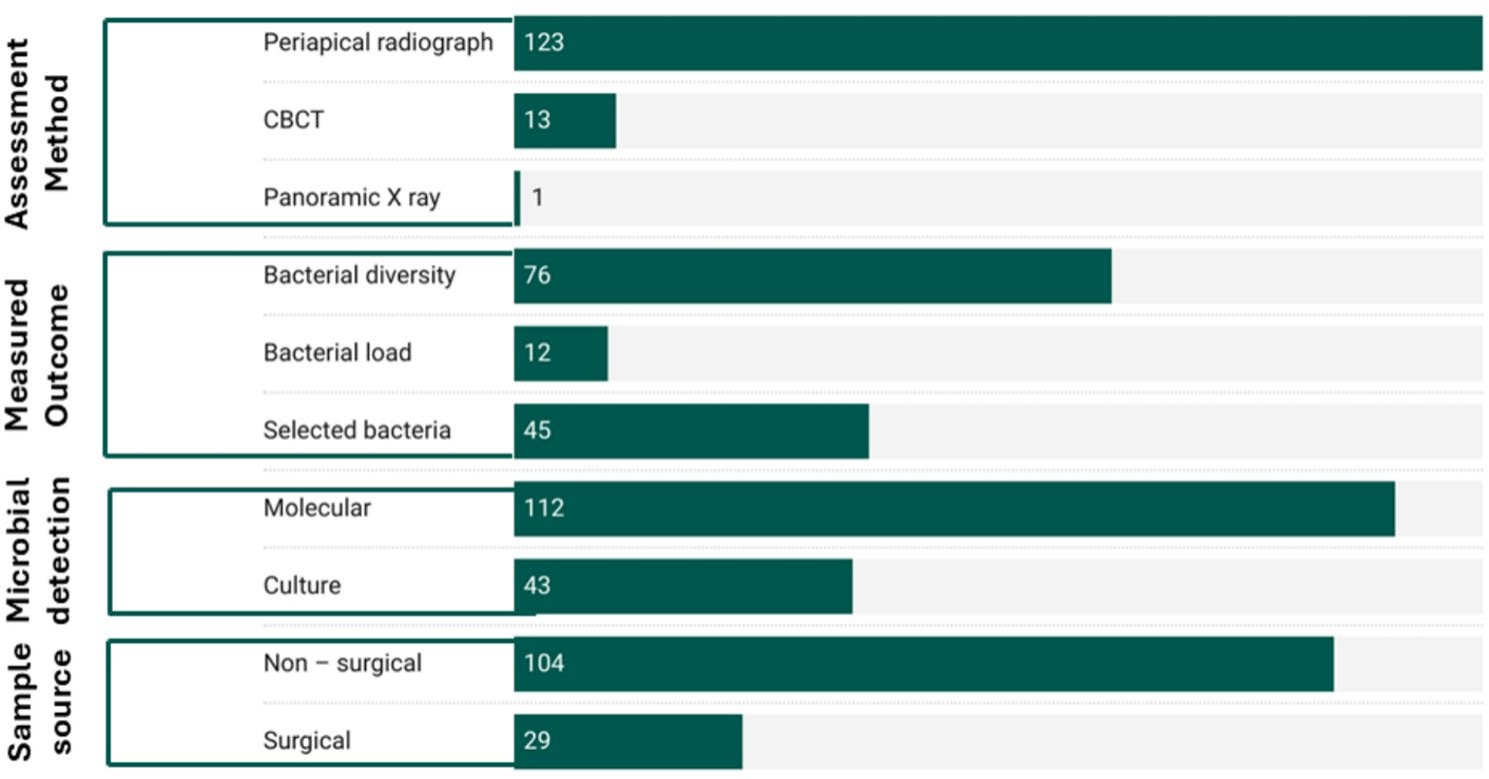
Outcome characteristics of the included studies

Molecular methods (e.g., PCR, DNA sequencing) were used in 85.5% of studies and culture-based methods were applied in 32.8% while both methods were used in 18.3% of the studies (Table 3).

Among the included studies, 123 (93.89%) used periapical radiographs, while only 13 studies (9.9%) used cone-beam computed tomography (CBCT) and only one study used Panoramic X-rays (Table 3).

### Microbial composition

Across the included studies, 64 microbial species were detected, with a predominance of anaerobic and facultative anaerobic bacteria. The most frequent taxa were *Fusobacterium* spp. (32.8%), *Streptococcus* spp. (29.8%), and *Enterococcus faecalis* (29%), forming the core endodontic bacteriome. Strict anaerobes (*P. gingivalis* 22.1%, *Prevotella* spp. 17.6%) and other pathogens (*P. endodontalis*, *T. denticola*, *T. forsythia*) were also prevalent. Occasional viral detection (Epstein Barr virus (EBV), human cytomegalovirus (HCMV), Herpes Simplex virus 1(HSV1), human papilloma virus (HPV), and human herpes virus-6 (HHV-6)) highlights the polymicrobial nature of these infections (Supplementary Table 6).

### Association between microbial diversity and the lesion size (Table 4)

Amongst the 132 studies, only 26.5% provided lesion size. An exploratory sub-analysis of approximately 26% of the included studies (*n* = 35) that filtered microbial data by lesion dimensions found that the endodontic microbiome composition was fairly constant across large and small lesions (Table 4). *Enterococcus faecalis* remained the most frequently reported species in both large (45.5%) and small (43.5%) lesions. Similarly, *Streptococcus* spp. (33.3% vs. 39.1%) and *Fusobacterium nucleatum* (30.3% vs. 30.4%) were present at comparable frequencies, supporting the notion that these core pathogens persist regardless of lesion size.

**Fig. 6:**
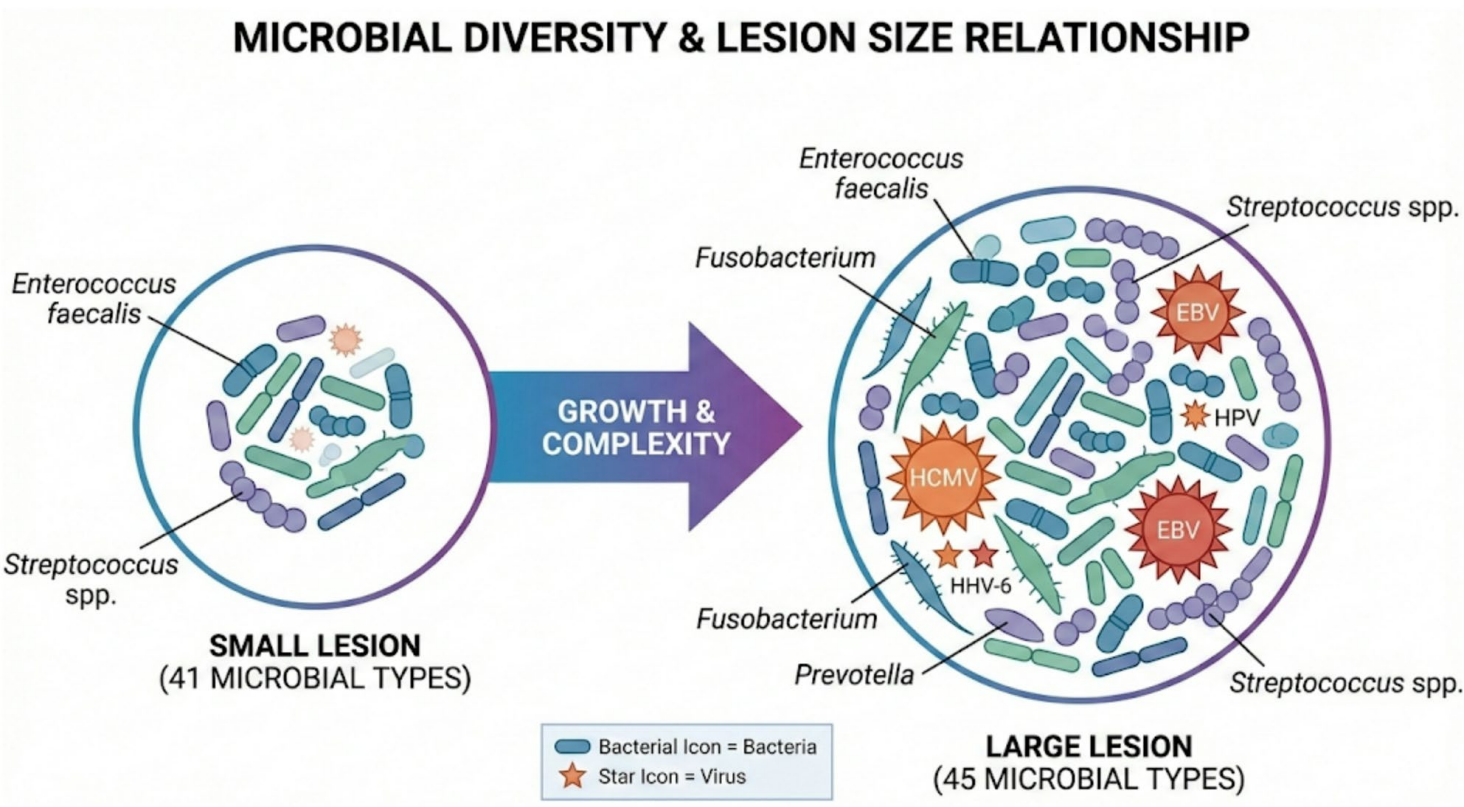
Microbial diversity and lesion size relationship

Certain bacteria (Olsenella, Lactococcus lactis) and viruses (HPV, HHV-6) were found to be exclusively present in larger lesions, implying that more chronic or expansive lesions may provide specific ecological niches for these organisms or involve viral co-factors in lesion progression (Fig. 6).

## Discussion

Understanding the association between microbial diversity and the size of apical lesions could guide clinical decision-making to enhance diagnosis, prognosis, treatment strategies, and improve outcomes of endodontic retreatments. Therefore, this scoping review was conducted to shed light on microbial diversity and its influence on the size of apical lesions in primary or secondary/persistent endodontic infections to clarify key concepts before undertaking more focused investigations [142].

### The inter-relationship between geographical location, systemic condition and microbiota in apical periodontitis

Studies comparing the microbiota of periapical infections in patients residing in different geographic locations revealed significant differences in the prevalence of certain pathogens [33, 40, 43, 46, 47, 53, 68, 86, 89, 125, 127, 129]. This may reflect differences in research funding, access to molecular diagnostics, or academic focus on the topic of endodontic microbiology. The number of publications remained mainly consistent highlighting the importance of studying microbial diversity in endodontic disease, which remained a key focus of studies over time. This geographic distribution of studies may also indicate that some microbial species may be more prevalent in certain countries particularly, demonstrating that the hosts’ geographical location, ethnicity, dietary and life styles may have an impact on microbial profiles [140]. This heterogeneity highlights the significance of analyzing the endodontic infection microbiome across distinct communities, even though it is unclear if it is a result of random variation or a reflection of genetic or environmental differences between different populations [10, 75].

It has been taken into consideration that only studies with a healthy population were selected in this scoping review, to avoid the influence of associated systemic diseases on bacterial diversity. Previous studies have shown that systemic diseases such as uncontrolled diabetes and autoimmune diseases may have a detrimental impact on periapical healing [66, 74, 143–146]. Interestingly, one of the studies included did not mention medical history as an eligibility criterion but rather stratified the patients later according to their ASA (American Society of Anesthesiologists) category [37, 147]. Indeed, this particular study correlated the medical history of the patients and microbial diversity detected via metagenomic testing and found that patients with medical history of systemic diseases had microbial constituents of higher richness and diversity. This further emphasizes the complex interrelationships between the endodontic microbiome and the host immune response [37, 147].

### Microbial detection methods could influence the interpretation of outcomes

Microbial detection methods in endodontic infections have evolved significantly, allowing for more accurate identification of the diverse microbiota involved in root canal infections. These methods can be broadly categorized into culture-based methods and molecular techniques [20, 30, 56, 57, 99, 101, 135]. The latter have identified probable novel endodontic pathogens, validated and reinforced the link between apical periodontitis and several uncultivable bacterial species such as Dialister invisus [53], Atopobium parvulum [34, 81] and HSV-1 virus [111, 134] as in the study conducted by Zargar et al. [36, 44]. They also offer highly sensitive and fast alternatives to traditional culturing for microbial detection and identification [21]. Sanchez-Sanhueza G et al. 2018 [37] reported that Next-generation sequencing (NGS) helped to reveal the bacterial composition and its relationship with the host.

In this scoping review, it could be observed that the detection of a large number of species was associated with molecular detection methods [22, 23, 115] of root canal microbiota despite one study showed that the percentage of identified phyla in culture was comparable to the results found with use of the molecular method [45].

### Other contributing factors that could influence microbial diversity

One of the strengths of this review is that it did not have any constraints on the type of teeth, patient age [148] or source of sampling as it is worth mentioning that these may be some of the factors influencing microbial diversity. Teeth with multiple root canals [19, 42, 65, 78, 112, 113, 116, 117, 149] showed an increased liability of developing periapical radiolucency (PR) and widened periodontal ligament space (PDL) and showing symptoms [50, 74, 88], in comparison to their single-rooted counterparts. Another factor is the difficult anatomical variations that lead to presence of unfilled root canal areas, fins and isthmuses, because it was discovered that the extent of the lesion and perhaps the number of bacteria present could be strongly correlated with the volume of unfilled apical canal regions [150–153]. Siqueira, JF et al. demonstrated that for a 1-unit increase in the percentage of unfilled apical canal system volume, the odds of large versus small lesions were 1.02 times greater. In other words, a unit increase in the amount of the unfilled apical canal system was associated with a 2% increase in the odds of large lesions compared with small lesions [38]. 

It was demonstrated that the type of endodontic infection, which is presented as primary and secondary infections, could affect microbial diversity [25, 26, 28, 34, 48, 93, 102, 120, 133] This review showed that primary infections were presented mostly (57.3%), followed by secondary infections (32.5%) in association with microbial diversity. It was reported that primary endodontic infections are often associated with higher number of bacterial cells [130, 154] than that found in secondary infection cases and that was linked with clinical symptoms as spontaneous pain, pain on palpation and pain to percussion [107]. This may be a result of the immune system’s reaction to the presence of microbes where polymicrobial flora as anaerobic gram-negative rods like Fusobacterium [82], Prevotella, and Porphyromonas species form the bulk of the population [59, 88, 149]. On the other hand, secondary or persistent infections tend to be more restricted in diversity [68, 131, 141]with prevalence of more resistant microorganisms like Candida albicans [72, 75, 96, 138] and Enterococcus faecalis [32, 60, 114, 136] which are adept at surviving in harsh environments. This is due to its potent virulence factors [94]. such as gelatinase, hyaluronidase, bacteriocins, aggregation substances, and lipoteichoic acid that were suggested to play an important role in the pathogenicity and biofilm formation. It has also been more linked to coronal microleakage [62] which is more associated with cases presented with chronic apical periodontitis [122]. Other microbiomes can be seen in both primary and secondary endodontic lesions such as T. denticola [57] and are correlated with radiographic periapical bone alterations [39].

### Inter-relationship between microbial species and clinical diagnosis/symptoms

Variations in the collection of samples and sample sources under the various clinical circumstances (such as acute apical abscesses or persistent infections) and in the procedures used for analysis may account for the variations in the microbiome system [77, 121, 126]. This indicates that the disease’s development is not caused by a single pathogen but rather by the community as a whole and its pathogenicity, which accounts for one of the causes of microbial diversity [55, 155].

The present scoping review showed a great diversity of microorganisms involved in endodontic infections [64, 79, 80, 103, 156, 157]. in most of the studies with studies assessing bacterial diversity accounting for 58% with high prevalence of Fusobacterium spp. (32.8%), Streptococcus spp. (29.8%) Enterococcus faecalis (29%), Porphyromonas gingivalis (22.1%), and Prevotella spp. (17.6%) among 59 identified microbial genera/species (Table 4). As mentioned by Lima A et al. [45] that this may be due to presence of the cnm+ gene which is a collagen (and laminin) binding protein. It appears that this collagen-binding protein gene plays an underestimated role in asymptomatic/chronic endodontic infections. Enterococcus faecalis, Porphyromonas gingivalis, and Prevotella spp. are frequently found in endodontic infections due to their ability to thrive in the anaerobic (oxygen-free) environment of the root canal, their resistance to some disinfection methods, and their role in biofilm formation [35, 100] while Viruses were reported in a minority of studies. It might be due to a historical focus on bacterial dominance over viruses in the field. In addition, techniques for detecting viruses in endodontic samples are less developed and may not be as sensitive or comprehensive as those used for bacteria [5]. 

Other studies evaluated specifically - selected bacteria [61, 97, 105, 141] including E. faecalis in addition to P. endodontalis, F. nucleatum, P. intermedia, T. forsythia, P. gingivalis, P. nigrescens, and T. denticola accounting for 34.4% of the studies and were presented in both large and small sized lesions. This was demonstrated by focused searching for the same microorganisms identified in all phases of endodontic retreatment, Enterococcus faecalis and Porphyromonas gingivalis were most frequently found, demonstrating their strong resistance to endodontic therapies [155].Gram-negative species, such as A. actinomycetemcomitans, F. nucleatum, P. gingivalis, T. forsythia, and T. denticola [139], were associated with higher percentage in large lesions than small ones as demonstrated by our results, also they were detected in symptomatic persistent or secondary infections following root canal treatment. This may be due to their primary virulence component (endotoxin) which explains the persistent nature of some endodontic infections [27, 51].

Viruses are also related to periapical lesions showing different sizes as reported in the results that viruses EBV, HCMV, HSV-1, and HPV each appearing in ≤ 3.1% of studies in this scoping review [52, 56, 84, 123]. It has been shown that some viruses such as Human cytomegalovirus (HCMV) and Epstein-Barr virus (EBV), both related to the herpesvirus family, can remain dormant for a long time in the salivary glands and mucosal surfaces which are the most common sites of reactivation [109, 126]. Indeed, they were seen with higher prevalence in large lesions than small ones with 15.2%, 12.1% respectively in large lesions and 4.3% each in small lesions. Makino K et al. [51] showed that EBV was detected in 78.1% of samples of periapical granulomas by real-time PCR. Candida albicans and HSV 1 Herpes simplex virus 1 were found only in small sized lesions in 4.3% and Zargar N et al., found that they had a higher prevalence rate in the teeth with PA lesions smaller than 5 mm [36]. Moreover, Ozbek et al. [52] reported that in 27% of large abscess lesions and 10% of small ones, HCMV DNA was found; in 18% of large and 10% of small abscess lesions, EBV was found. 9% of large abscess lesions had HPV and HHV-6 DNA, while none of the small abscess lesions had either of these. It could be explained that viruses might affect the growth and progression of periapical lesions directly due to viral infection and replication that leads to generation of oxidative stresses [158] and elicits the production of cytokines [159] as mentioned by akovljevic et al. [160].

EBV positive periapical lesions displayed significantly higher mRNA expression of bone resorption regulators receptor activator of nuclear factor (NF-kB) ligand (RANKL) and osteoprotegerin (OPG) with an imbalance in their ratio compared to normal healthy tissues [161] or it may indirectly cause periapical lesion aggravation through impairment of the host immunity and defense systems as HCMV reactivation stimulates transactivation of EBV at the site of inflammatory reaction [162], or indirectly though weakening of the host immunity and defense systems and thus increasing the bacterial pathogenicity [123].

As an outcome, this association is proportional: as the size of the lesion increases, so does the complexity of the microbial community, particularly the prevalence of viral co-pathogens. Large lesions exhibited a viral spike, increasing the frequency of viruses where HCMV increases more than 3.5 times (from 4.3% to 15.2%) and EBV nearly triples (from 4.3% to 12.1%) in larger lesions. Additionally, large lesions were uniquely related to the presence of HPV (6.1%).

An intriguing observation affecting microbial diversity and thus affecting lesion size is that it varied according to the symptomatology [118, 139] as seen in this scoping review where asymptomatic apical periodontitis (AAP) comprised (80.5%) and symptomatic apical periodontitis (SAP) (50.6%). The known virulence factors; lipopolysaccharide and lipoteichoic acid can be related to clinical symptoms [21]. Some pathogens like P. gingivalis, T. denticola and T. forsythia (“red complex”) which demonstrated higher percentages in the recorded microbiomes in the present review (22.2%, 12.20%, 10.7% respectively) and had an association with both large and small lesion sizes have been shown to cause pain, tissue swelling, and sinus tracts and to be more associated with symptomatic apical periodontitis [54, 109, 163]. Also F. nucleatum which showed the highest percentage in the recorded microbiomes and showed association with both large and small sized lesions with nearly equal percentages appears to be more abundant in symptomatic lesions which suggests that it may be the reason behind inflammation that leads to severe tissue damage [83].

### Limitations and possible knowledge gaps

A significant obstacle was found when conducting the current review while reporting periapical lesion sizes or dimensions that only (26.7%) of the studies mentioned lesion size. It is important that future studies focus on accurately documenting apical lesion size, particularly when microbial analysis is to be executed. Furthermore, most studies used 2D periapical radiographs while the use of CBCT was more common in more recent studies. The added advantage of 3 dimensions in CBCT provides further accuracy in determining lesion size [12, 93]. Studies included in the current review used periapical radiographs in 93.9% of studies, while CBCT analysis was used in only in 9.9% and that could be attributed to the degree of accessibility, financial reasons and the constraints about radiation doses. Additionally, standardizing how radiographic outcomes are measured is another critical aspect. In this review, it was attempted to correlate known indices with numerical measurements to approximate the readings of the PAI or the CBCTPAVI indices to numerical readings of more or less than 5 mm and vice versa to help reach a consensus about reporting lesion dimensions.

Microbial load is another major determinant of periapical lesion size [90, 92, 108, 164] with anaerobic, polymicrobial infections causing the most destruction [24, 69, 70, 95, 124, 132]. Large lesions may show a higher number of species while not differing significantly from small ones in bacterial diversity [18, 29, 49, 165], upon this an assumption was made that large lesions might be a factor of time and that larger lesions are supposed to be older ones. This can be explained in the context of the longer the time the lesion present, the higher the bacterial load and higher probability of more bacterial species to proliferate [1, 38, 166–168] but this was contradicted based on our findings. Primary periapical lesions and lesions due to pulp necrosis which formed the higher percentage of the included studies (67%, 71% respectively) showed larger sizes with greater microbial diversity as this might be a factor of bacterial virulence that affects the microbial community and guides its aggressiveness due to the synergistic relation between different microbiomes [31, 102, 169] Additionally, Siqueira, JF et al. [38] did not find any significant associations between the apical canal’s overall bacterial count and the time elapsed since treatment/retreatment. A probable reason might also be due to the high interindividual variability [18].

The proof of adherence to the recently revised JBI scoping review guidelines is a major strength of this scoping review [170, 171]. Additionally, this is the first scoping review that we are aware of that addresses the specific question of how microbial diversity influences the size of periapical lesions in primary or secondary/persistent endodontic infections. On the other hand, some limitations were found related to the heterogeneity of the sampling methods. Concerning the molecular methods of microbial detection, it must be taken into consideration that the sensitivity and specificity of microbiological techniques used to identify microorganisms may affect the results. Also, the lack of unifying the radiographic analysis of lesion size either with measurements or depending on indices led to difficulty in the exact accurate determination of lesion size. Although this scoping review focuses on clinical studies, only 15 out of 131 studies were identified as randomized clinical studies. The lack of studies that followed the CONSORT guidelines may lead to a lack of generalizability of the results. There was a lack of risk of bias assessment in this review as its objective was to broadly map the existing literature not critically evaluate evidence’s validity.

Significant knowledge gaps were also identified by this review and recommendations, including the importance of studying microbial richness and diversity to provide a more accurate range of pathogens to select antimicrobial agents in root canal treatment are suggested. Moreover, future longitudinal clinical studies employing metagenomic sequencing and volumetric CBCT analysis of lesions are needed. More clinical studies for periradicular tissue markers to indicate healing or not are also recommended. Further studies should also consider virulence factors and pathogenicity of certain bacterial strains especially F. nucleatum, A. rimae, D. invisus, and M. osloensis. and endotoxin which plays an important role in pathogenicity, biofilm formation and expression of apical periodontitis.

## Conclusions

Within the limitations of this scoping review and taking into consideration the small number of studies that mentioned apical lesion size it was concluded that Microbial diversity and microbial load seem to be a strong determinant of apical lesion size while lesion duration could not be adequately assessed due to cross-sectional study designs. Lesion size is an important variant to be recorded to give insight into microbial diversity and provide the basis for personalized targeted antimicrobial therapies in the future.Among various types of microorganisms, Fusobacterium spp., Streptococcus spp., Enterococcus faecalis, Porphyromonas gingivalis and Prevotella spp were the most common bacteria in microbial community related to endodontic infections.

## Supplementary Information

Supplementary Material 1.

## Data Availability

No datasets were generated or analysed during the current study.

## Abbreviations

AP: Apical periodontitis
PAP: Persistent apical periodontitis
SAP: Symptomatic apical periodontitis
AAP: Asymptomatic apical periodontitis
CBCT: Cone-beam Computed tomography
PR: Periapical radiolucency
LTA: Lipot eichoic acid
EBV: Epstein Barr virus
HCMV: Human cytomegalovirus
HPV: Human papilloma virus
HSV: Human simplex virus
HHV: Human herpes virus
ASA: American Society of Anesthesiologists
Cnm: Collagen-binding protein Cnm for streptococcus mutans strains
