# Supplementary material for: Is apical periodontitis a matter of microbial diversity or time? A scoping review
Source: Head Face Med. 2026 Mar 31;22:42. doi: 10.1186/s13005-026-00610-4 (PMC13085567; doi:10.1186/s13005-026-00610-4)
Supplement: Supplementary file 1 — Supplementary Material 1. [file 13005_2026_610_MOESM1_ESM.docx]

**Supplementary table 1: Risk of Bias for included RCTs**

| Study ID | Q1 | Q2 | Q3 | Q4 | Q5 | Q6 | Q7 | Q8 | Q9 | Q10 | Q11 | Q12 | Q13 | Overall Risk |
| --- | --- | --- | --- | --- | --- | --- | --- | --- | --- | --- | --- | --- | --- | --- |
| Amaral RR et al, 2022 | Yes | Unclear | Yes | No | No | Yes | Yes | Yes | Yes | Yes | Yes | Yes | Yes | Moderate |
| Cavalli D et al, 2017 | Yes | Unclear | Yes | No | No | Yes | Yes | Yes | Yes | Yes | Yes | Yes | Yes | Moderate |
| de Miranda RG et al, 2018 | Yes | Unclear | Yes | No | No | Unclear | Yes | Yes | Yes | Yes | Yes | Yes | Yes | Moderate |
| Ersahan S et al, 2022 | Yes | Unclear | Yes | No | No | Yes | Yes | Yes | Yes | Yes | Yes | Yes | Yes | Moderate |
| Hepsenoglu YE et al, 2023 | Yes | Unclear | Yes | No | No | Yes | Yes | Yes | Yes | Yes | Yes | Yes | Yes | Moderate |
| Kist S et al, 2017 | Yes | Unclear | Yes | No | No | Yes | Yes | Yes | Yes | Yes | Yes | Yes | Yes | Moderate |
| Moghadam MD et al, 2021 | Yes | Unclear | Yes | No | No | Yes | Yes | Yes | Yes | Yes | Yes | Yes | Yes | Moderate |
| Orozco E et al, 2020 | Yes | Unclear | Yes | No | No | Yes | Yes | Yes | Yes | Yes | Yes | Yes | Yes | Moderate |
| Pelozo LL et al, 2023 | Yes | Unclear | Yes | No | No | Yes | Yes | Yes | Yes | Yes | Yes | Yes | Yes | Moderate |
| Ping Y et al, 2015 | Yes | Unclear | Yes | No | No | Yes | Yes | Yes | Yes | Yes | Yes | Yes | Yes | Moderate |
| Stojanović N et al, 2014 | Yes | Unclear | Yes | No | No | Yes | Yes | Yes | Yes | Yes | Yes | Yes | Yes | Moderate |
| Toia CC et al, 2022 | Yes | Unclear | Yes | No | No | Yes | Yes | Yes | Yes | Yes | Yes | Yes | Yes | Moderate |
| Zahran S et al, 2021 | Yes | Unclear | Yes | No | No | Yes | Yes | Yes | Yes | Yes | Yes | Yes | Yes | Moderate |
| Zahran S et al, 2022 | Yes | Unclear | Yes | No | No | Yes | Yes | Yes | Yes | Yes | Yes | Yes | Yes | Moderate |
| Zandi H 2019 | Yes | Unclear | Yes | No | No | Yes | Yes | Yes | Yes | Yes | Yes | Yes | Yes | Moderate |

**Supplementary Table 2:** **Risk of Bias for included Quasi experimental studies**

| Study ID | Q1 | Q2 | Q3 (Control) | Q4 | Q5 | Q6 | Q7 | Q8 | Overall Risk |
| --- | --- | --- | --- | --- | --- | --- | --- | --- | --- |
| Alquria TA et al, 2024 | Yes | Yes | No | Yes | Yes | Yes | Yes | Yes | Moderate |
| Card SJ et al, 2002 | Yes | Yes | No | Yes | Yes | Yes | Yes | Yes | Moderate |
| Garcez AS et al, 2015 | Yes | Yes | No | Yes | Yes | Yes | Yes | Yes | Moderate |
| Nogales CG et al, 2025 | Yes | Yes | No | Yes | Yes | Yes | Yes | Yes | Moderate |
| Waltimo T et al, 2005 | Yes | Yes | No | Yes | Yes | Yes | Yes | Yes | Moderate |

**Supplementary Table 3:** **Risk of Bias for included observational studies**

| Study ID | Q1 | Q2 | Q3 (Method) | Q4 | Q5 (Conf.) | Q6 (Mgmt) | Q7 | Q8 | Overall Risk |
| --- | --- | --- | --- | --- | --- | --- | --- | --- | --- |
| Abushouk S et al, 2021 | Yes | Yes | Yes | Yes | No | No | Yes | Yes | Moderate |
| Ahmed S et al, 2024 | Yes | Yes | No | Yes | No | No | Yes | Yes | Moderate |
| Alquria T et al, 2024 | Yes | Yes | Yes | Yes | Yes | Yes | Yes | Yes | Low |
| Andric M et al, 2007 | Yes | Yes | Yes | Yes | No | No | Yes | Yes | Moderate |
| Arias‐Moliz MT et al, 2024 | Yes | Yes | No | Yes | No | No | Yes | Yes | Moderate |
| Barani M et al, 2023 | Yes | Yes | No | Yes | No | No | Yes | Yes | Moderate |
| Blome B et al, 2008 | Yes | Yes | Yes | Yes | No | No | Yes | Yes | Moderate |
| Bogen G & Slots J, 1999 | Yes | Yes | Yes | Yes | No | No | Yes | Yes | Moderate |
| Brenda P. F. A et al, 2021 | Yes | Yes | Yes | Yes | No | No | Yes | Yes | Moderate |
| Bronzato JD et al, 2021 | Yes | Yes | Yes | Yes | Yes | Yes | Yes | Yes | Low |
| Buonavoglia A et al, 2023 | Yes | Yes | Yes | Yes | No | No | Yes | Yes | Moderate |
| Cao H et al, 2012 | Yes | Yes | Yes | Yes | Yes | Yes | Yes | Yes | Low |
| Cardoso FG et al, 2016 | Yes | Yes | Yes | Yes | Yes | Yes | Yes | Yes | Low |
| Carvalho AP et al, 2020 | Yes | Yes | Yes | Yes | No | No | Yes | Yes | Moderate |
| Didilescu AC et al, 2012 | Yes | Yes | Yes | Yes | No | No | Yes | Yes | Moderate |
| Donnermeyer D et al, 2025 | Yes | Yes | Yes | Yes | No | No | Yes | Yes | Moderate |
| Foschi F et al, 2005 | Yes | Yes | Yes | Yes | No | No | Yes | Yes | Moderate |
| Geibel M et al, 2005 | Yes | Yes | Yes | Yes | No | No | Yes | Yes | Moderate |
| Godoi-Jr EP et al, 2023 | Yes | Yes | Yes | Yes | Yes | Yes | Yes | Yes | Low |
| Gomes BP et al, 2020 | Yes | Yes | Yes | Yes | Yes | Yes | Yes | Yes | Low |
| Gomes BP et al, 2021 | Yes | Yes | Yes | Yes | No | No | Yes | Yes | Moderate |
| Gomes BPFA et al, 2005 | Yes | Yes | Yes | Yes | No | No | Yes | Yes | Moderate |
| Gomes BPFA et al, 2006 | Yes | Yes | Yes | Yes | No | No | Yes | Yes | Moderate |
| Gomes BPFA et al, 2007 | Yes | Yes | Yes | Yes | Yes | Yes | Yes | Yes | Low |
| Gomes BPFA et al, 2015 | Yes | Yes | Yes | Yes | No | No | Yes | Yes | Moderate |
| Henriques LC et al, 2016 | Yes | Yes | Yes | Yes | No | No | Yes | Yes | Moderate |
| Hou Y et al, 2021 | Yes | Yes | Yes | Yes | No | No | Yes | Yes | Moderate |
| Hu Z et al, 2023 | Yes | Yes | Yes | Yes | Yes | Yes | Yes | Yes | Low |
| Jacinto RC et al, 2008 | Yes | Yes | No | Yes | Yes | Yes | Yes | Yes | Moderate |
| Machado et al, 2020 | Yes | Yes | Yes | Yes | Yes | Yes | Yes | Yes | Low |
| Makino K et al, 2015 | Yes | Yes | Yes | Yes | No | No | Yes | Yes | Moderate |
| Martinho FC et al, 2010 | Yes | Yes | Yes | Yes | Yes | Yes | Yes | Yes | Low |
| Murad CF et al, 2014 | Yes | Yes | Yes | Yes | Yes | Yes | Yes | Yes | Low |
| Nobrega LM et al, 2016 | Yes | Yes | Yes | Yes | Yes | Yes | Yes | Yes | Low |
| Ozbek SM et al, 2016 | Yes | Yes | Yes | Yes | No | No | Yes | Yes | Moderate |
| Pérez‐Carrasco V et al, 2023 | Yes | Yes | Yes | Yes | Yes | Yes | Yes | Yes | Low |
| Ribeiro AC et al, 2011 | Yes | Yes | Yes | Yes | No | No | Yes | Yes | Moderate |
| Sabeti M & Slots J, 2004 | Yes | Yes | No | Yes | No | No | Yes | Yes | Moderate |
| Sanghavi TH et al, 2014 | Yes | Yes | Yes | Yes | No | No | Yes | Yes | Moderate |
| Sassone L et al, 2007 | Yes | Yes | Yes | Yes | Yes | Yes | Yes | Yes | Low |
| Sassone LM et al, 2008 | Yes | Yes | Yes | Yes | Yes | Yes | Yes | Yes | Low |
| Sedgley C et al, 2006 | Yes | Yes | Yes | Yes | No | No | Yes | Yes | Moderate |
| Siqueira JF & ROCAS, 2002 | Yes | Yes | Yes | Yes | No | No | Yes | Yes | Moderate |
| Siqueira JF et al, 2016 | Yes | Yes | Yes | Yes | No | No | Yes | Yes | Moderate |
| Sun X et al, 2022 | Yes | Yes | Yes | Yes | Yes | Yes | Yes | Yes | Low |
| Sunde PT et al, 2001 | Yes | Yes | Yes | Yes | No | No | Yes | Yes | Moderate |
| Tiwari S et al, 2020 | Yes | Yes | Yes | Yes | No | No | Yes | Yes | Moderate |
| Zargar N et al, 2019 | Yes | Yes | Yes | Yes | Yes | Yes | Yes | Yes | Low |
| Zargar N et al, 2020 | Yes | Yes | Yes | Yes | Yes | Yes | Yes | Yes | Low |
| Zakaria MN et al, 2015 | Yes | Yes | Yes | Yes | Yes | Yes | Yes | Yes | Low |

**Supplementary Table 4: frequency and percentage of bacteria and viruses**

| Bacteria | Frequency | % |
| --- | --- | --- |
| Fusobacteria (Fusobacterium Spp (F. Nucleatum, Necrophorum)) | 43 | 32.8 |
| Streptococcus Spp. (S. Constellatus, S. Intermedius, Streptococcus Sanguis, Salivarius, Mutans, Mitis, Oralis) | 39 | 29.8 |
| Enterococcus Faecalis | 38 | 29 |
| Porphyromonas Gingivalis | 29 | 22.1 |
| Prevotella Spp (Intermedia, Nigrescens, Loescheii) | 23 | 17.6 |
| Porphyromonas (Endodontalis) | 17 | 13 |
| Treponema Denticola | 16 | 12.20 |
| Bacteroidetes Spp | 15 | 11.5 |
| Peptostreptococcus Spp | 15 | 11.5 |
| Tannerella Forsythia | 14 | 10.7 |
| Parvimonas Micra | 14 | 10.7 |
| Actinobacteria (Actinomyces) | 12 | 9.2 |
| Firmicutes | 11 | 8.4 |
| Staphylococcus Epidermidis | 11 | 8.4 |
| Proteobacteria | 10 | 7.6 |
| Veillonella | 10 | 7.6 |
| Actinomyces Naeslundii | 9 | 6.9 |
| Synergistetes | 9 | 6.9 |
| Dialister Invisus | 7 | 5.3 |
| Candida Albicans | 7 | 5.3 |
| Capnocytophaga (Capnocytophaga Sputigena | 6 | 4.6 |
| Lactobacillus Rhamnosus | 6 | 4.6 |
| Dialister Pneumosintes | 6 | 4.6 |
| Campylobacter Rectus | 6 | 4.6 |
| Lactobacillus Gasseri | 5 | 3.8 |
| Gemella Morbillorum | 5 | 3.8 |
| Actinomyces Israelii | 5 | 3.8 |
| Porphyromonas Genera | 4 | 3.1 |
| Atopobium Rimae | 4 | 3.1 |
| Filifactor Alocis | 4 | 3.1 |
| Pseudomonas Gessardii | 4 | 3.1 |
| EBV | 4 | 3.1 |
| HCMV | 4 | 3.1 |
| Aerococcus Viridans | 3 | 2.3 |
| P. Melaninogenica | 3 | 2.3 |
| Leptotrichia | 3 | 2.3 |
| Neisseria Spp. | 3 | 2.3 |
| Pseudoramibacter Alactolyticus | 3 | 2.3 |
| Eubacterium Lentum | 3 | 2.3 |
| Propionibacterium Acnes | 3 | 2.3 |
| Spirochaetes | 2 | 1.5 |
| Burkholderia | 2 | 1.5 |
| Bifidobacterium | 2 | 1.5 |
| Aggregatibacter Actinomycetemcomitans | 2 | 1.5 |
| E. Faecium | 2 | 1.5 |
| HSV 1 | 2 | 1.5 |
| Lactococcus Lacti | 1 | 0.8 |
| Morganella | 1 | 0.8 |
| Solobacterium Moorei | 1 | 0.8 |
| Vagococcus Fluvialis | 1 | 0.8 |
| Micromonas Micros | 1 | 0.8 |
| Lysinibacillus Fusiformis | 1 | 0.8 |
| Haemophilus | 1 | 0.8 |
| Lactobacillus Spp | 1 | 0.8 |
| HPV | 1 | 0.8 |
| HHV-6 | 1 | 0.8 |
| Granulicatella Elegans | 1 | 0.8 |
| Clostridium Bifermentans | 1 | 0.8 |
| Corynebacterium Diphtheriae | 1 | 0.8 |
| Stenotrophomonas Maltophilia | 1 | 0.8 |
| Rodotorula Mucilaginosa | 1 | 0.8 |
| Archae (Methanobrevibacter | 1 | 0.8 |
| Pasteurellales, Vibrionales | 1 | 0.8 |
| Olsenella | 1 | 0.8 |

**Supplementary Table 5: Microbial diversity and load in the included studies**

| **author** | **microbial detection method** | **source of the sample /surgical or non-surgical** | **Topmost commonly found microorganisms in infection site** | **Topmost commonly found microorganisms after final sampling** | **Microbial load/ reduction** | **Follow up using radiograph** |
| --- | --- | --- | --- | --- | --- | --- |
| Amaral R R et al, 2022 | molecular based | non-surgical, main root canal | Most common microbiome: Firmicutes (27%), Bacteroidetes (21%), Proteobacteria (21%), and Actinobacteria (12%). | NA | Microbial load: a very high bacterial diversity in the microbiome of teeth with asymptomatic apical periodontitis, 4 phyla dominated the microbiome: Firmicutes (27%), Bacteroidetes (21%), Proteobacteria (21%), and Actinobacteria (12%). Large lesions showed a higher number of species but did not significantly differ from small lesions in bacterial diversity indexes. | NA |
| Handal T et al, 2009 | molecular based | surgical, root apices | Most common microbiome: Fusobacterium spp., Prevotella spp., Tannerella forsythia, Porphyromonas endodontalis, Treponema denticola, Bacteroidetes spp., Peptostreptococcus spp., and Streptococcus spp. | NA | Microbial load: A high bacterial diversity, with 75 different bacterial taxa, were identified amongst the 236 clones investigated | NA |
| Rodríguez R et al, 2023 | Culture-Based | Non-surgical, main root canal | Most common microbiome: Actinomyces naeslundii 23.61 %, Enterococcus faecalis 16.66 %, Aerococcus viridans 8.33 %, Streptococcus sanguis 6.94 %, Fusobacterium nucleatum 6.94 % | Most common microbiome: Actinomyces naeslundii (40.00%), Enterococcus faecalis (22.85%), Aerococcus viridans 1(1.42%), Porphyromona asaccharolytica (5.71%), Fusobacterium nucleatum (2.85%) (after medication) | Microbial reduction: The quantification of CFU counting showed a bacterial decrease between the pre-instrumentation, post-instrumentation, and post-medication samples, with a statistically significant difference between all groups, which means that each step procedure contributed to the control of the odontogenic infection. | CBCT: Periapical Lesions were evaluated by CBCT after six months of endodontic treatment. According to the data obtained, there was a decrease in the volume size of the periapical lesion |
| Orozco E et al, 2020 | culture-based | Non-surgical, main root canal | Most common microbiome: S. constellatus (50%), E. faecalis (45%), F. nucleatum SP (45%), P. gingivalis (45%), P. melaninogenica (45%) and S. intermedius (45%) | E. faecalis (50%) the most frequent species | Microbial reduction: No statistical difference in the number of species detected or the total bacterial load between S2 and S3 (p>0.05) was observed. The mean number of bacterial species in S3 was 7.6±5.5 and 9.8±6.3, respectively, | CBCT: After 18 months follow up, the reduction was detected for both treatments (p<0.043), |
| Ping Y et al, 2015 | molecular based | surgical, root apices | Most common microbiome: The abundance of seven phyla; Firmicutes (31%), Proteobacteria (23%), Bacteroidetes (19%), Fusobacteria (12%), Actinobacteria (10%), Synergistetes (2%), and Spirochaetes (2%). The abundance genera: Streptococcaceae_Streptococcus (12%), Burkholderia (8%), Prevotella (7%), Fusobacterium (7%), Veillonella (5%), Leptotrichia (5%), Capnocytophaga (4%), and Actinomyces (4%). | NA | Microbial load: Massive interindividual variations were revealed in the components of apical microbiota. Pyrosequencing technique helped to discover an unexpectedly high bacteria diversity of apical microbiota in this study Compared with the traditional molecular biological methods. | NA |
| Zahran S et al, 2022 | molecular based | non-surgical, main root canal | NA | Most common microbiome: in Standard protocol S2 samples; Bacteroidetes (B. bacterium HMT 365), Fusobacterium (F. nucleatum subsp. animalis) , Peptostreptococcaceae ( yurii subspp. yurii & margaretiae ) , Porphyromonas (P. pasteri ) , and Veillonella (V. rogosae and V. parvula). In the Enhanced protocol S2 samples (Lower relative abundance), such as Actinomyces (A. sp. HMT 1172,A. naeslundii , A. sp. HMT 180) , Haemophilus (H. parainfluenzae ) , Cutibacterium ((CC.nes) , and Prevotella ( P. Pallens) | Microbial reduction: After instrumentation and irrigation, the mean number of detectable OTUs per sample was reduced to 38 and 26 in the pre-canal filling samples of Standard and Enhanced protocols, respectively. | NA |
| Zahran S et al, 2021 | molecular based | non-surgical, main root canal | NA | NA | Microbial reduction: The enhanced protocol significantly reduced bacterial counts in pre-canal filling samples when compared to the standard protocol. | CBCT: outcomes assessed by CBCT was 85.2% in the enhanced infection control protocol and 66.7% in the standard protocol after one-year follow-up. |
| Zandi H 2019 | molecular based | non-surgical, main root canal | NA | NA | Microbial reduction: In both the healed and not healed groups, the mean total bacterial counts reduced significantly from S1(immediately after root canal filling removal for retreatment) to S2 (after instrumentation using irrigant) and from S1 to S3 (after removal of the interappointment calcium hydroxide dressing) (P < .01). The bacterial load in S3 was significantly higher in the not healed cases than in the healed cases. Microbial load: Large lesions harbored significantly higher bacterial counts than small lesions (P < .01). However, the lesion size did not influence the retreatment outcome (P > .05). | Periapical radiograph: After 1 year, 65% in the NaOCl group and 64% in the CHX group healed, with no differences between them ( P > .05).  At the later follow-up, the healing rates were 81% and 82%, respectively ( P > .05). |
| Korona-Glowniak I et al, 2021 | culture and molecular based | non-surgical, main root canal | Most common microbiomes: Firmicutes (62.9%), Actinobacteria (14.0%), Bacteroidetes (12.1%), Proteobacteria (9.1%) and Fusobacteria (4.2%) ,54.6% were strict anaerobe. Enterococcus faecalis and Candida albicans detected treatment-resistant infections. | NA | NA | NA |
| Sun X et al, 2022 | molecular based | non-surgical, surgical from periapical soft lesions | Most common microbiomes: Proteobacteria (31.5%), Firmicutes (20.9%), Bacteroidetes (13.2%), Actinobacteria (10.5%) represented the core microbiome of extraradicular and intraradicular infections associated with persistent apical periodontitis.At genus level: Fusobacteria (10.4%), Fusobacterium (with a mean relative abundance of 9.8%), Morganella (9.1%), Burkholderia (5.4%), Porphyromonas (5.3%), Streptococcus (4.8%), and Bifidobacterium (3.7%). In sinus tract: Porphyromonas, Eubacterium, Treponema, Phocaeicola represented the core microbiome of extraradicular and intraradicular infections associated with persistent apical periodontitis. | NA | Microbial load: samples from the extraradicular infection sites (i.e., periapical lesion samples and extraradicular biofilm samples) exhibited significantly higher OTU richness and a higher number of total OTUs than the samples from intraradicular infection site (i.e., root canal filling samples). | NA |
| Pérez‐Carrasco V et al, 2023 | molecular based | surgical, root apices | Most common microbiome: In the apices, Fusobacterium was the most abundant, followed by Streptococcus, Porphyromonas, Enterococcus, Pseudomonas and Tannerella, whereas Porphyromonas followed by Fusobacterium, Streptococcus, Fretibacterium and Pseudomonas were dominant in the periapical lesions. | NA | Microbial load: A higher alpha- diversity was observed in the periapical lesions, although no global differences in the community composition between the two sites were found | NA |
| Tennert C et al, 2014 | culture and molecular based | non-surgical main root canal | Most common microbiome: Teeth with insufficiently filled root canals and asymptomatic apical periodontitis (E . faecalis and Streptococcus mutans were isolated most frequently (19%). Lactobacillus rhamnosus , Parvimonas micra , and A . rimae in 9.5% of the cases. invisus,ccus mitis , Streptococcus sanguis , Streptococcus constellatus , Lactobacillus gasseri , Actinomyces naeslutimidum, osloensis , Eikenella corrodens , A. viscosus , A. prevotii , Bifidobacterium sp., D. invisus , Dialister pneumosintes , Veillonella dispar, Mogibacterium pumilum , Prevotella intermedia, Mogibacterium timidum , Filifactor alocis , Tannerella forsythia, Porphyromonas gingivalis , Campylobacter rectus, and Propionibacterium acidifaciens were isolated in 4.7% of the cases.). Teeth with chronic apical abscess: Atopobium rimae, Anaerococcus prevotii , Pseudoramibacter alactolyticus , Dialister invisus and Fusobacterium nucleatum. | After intracanal dressing with calcium hydroxide: A monoinfection of E . faecalis. After intracanal dressing with Ledermix: monoinfection of A . viscosus. T | NA | NA |
| Toia CC et al, 2022 | culture and molecular based | non-surgical main root canal | NA | NA | Microbial reduction: The 1-visit group showed a significantly lower microbial load compared with the 2-visit group. | CBCT: Both groups exhibited a statistically significant reduction in periapical lesion volume between the preoperative examination and 18-month evaluation ( P < .05), with no statistical differences between the groups |
| Shuping GB et al, 2000 | Culture Based | non-surgical main root canal | NA | NA | Microbial reduction: Instrumentation with NaOCl irrigation was superior in bacterial reduction to instrumentation with sterile saline with 61.9% of canals becoming free of bacteria. The addition of calcium hydroxide produced 92.5% of canals void of bacteria. | NA |
| Arias‐Moliz MT et al, 2024 | molecular based | surgical root apices and periapical soft lesions | Most common microbiome: Fusobacterium nucleatum (19.8%), Prevotella loescheii (4.18%), Streptococcus intermedius (4.17%), P. gingivalis (4.06%), Parvimonas micra (3.9%), Synergistetes bacterium (3.37%), Tannerella forsythia (3.32%), Peptostreptococcus stomatis (3.29%), Pseudomonas gessardii (2.72%) and Pseudoramibacter alactolyticus (2.2%)" | NA | Microbial load: A similar alpha-diversity (p > .05) was observed for apices and lesion samples . No differences were found in the community composition according to principal coordinate analysis (PCoA) between the two groups at OTU level | NA |
| Godoi-Jr EP et al, 2023 | Culture and molecular based | non-surgical main root canal | Most common microbiome: (1) Teeth indicated for endodontic retreatment due to the presence of chronic apical periodontitis (G1); -Before CMP (S1) inside the root canal of cases associated with the presence of periapical lesions were Enterococcus faecalis (86.6%), Fusobacterium nucleatum (66.6%), Porphyromonas gingivalis (66.6%), Parvimonas micra (33.3%), and Aggregatibacter actinomycetemcomitans (33.3%).(2) Teeth indicated for endodontic retreatment due to prosthetic reasons (G2); After root canal filling removal were Enterococcus faecalis (86.6%), Porphyromonas gingivalis (20%), Fusobacterium nucleatum (13.3%), and Aggregatibacter actinomycetemcomitans (13.3%). | (1) Teeth indicated for endodontic retreatment due to the presence of chronic apical periodontitis (G1): After the use of intracanal medication, Enterococcus faecalis detected (60%), Porphyromonas gingivalis (40%), Fusobacterium nucleatum (40%), and Aggregatibacter actinomycetemcomitans (13.3%). Prevotella tannerae, Actinomyces naeslundii, and Gemella morbillorum were no longer identified. (2) Teeth indicated for endodontic retreatment due to prosthetic reasons (G2): After intracanal medication, Enterococcus faecalis (53.3%), Porphyromonas gingivalis (6.6%), and Fusobacterium nucleatum (6.6%). | Microbial load: the microbial concentration present in cases with apical periodontitis was higher than those found in cases indicated for retreatment due to prosthetic reasons. After performing the CMP (S2) and the use of intracanal medication (S3), no statistically significant differences were observed between the bacterial load detected in both groups. | NA |
| Barbosa-Ribeiro M et al, 2020 | molecular based | non-surgical main root canal | Most common microbiome: E. faecalis (35.3%), Staphylococcus epidermidis (10.7%), Streptococcus sanguis (6.1%) and A. viridans (6.1%). before CMP | E. faecalis, the most prevalent species (33.3%) from 9 strains remained. | Microbial reduction: A reduction of 76.9% in the microbial content after CMP (S2) was observed. ICM allowed for an additional reduction of 9.3% in comparison with S2. After all endodontic procedures, the bacterial reduction was 86.2%. | Periapical radiograph: E. faecalis, F. nucleatum and P. gingivalis were associated with periapical lesion > 3 mm. |
| Schirrmeister JF et al, 2009 | molecular based | non-surgical main root canal | most common microbiome: Solobacterium moorei and F. nucleatum were the most prevalent species. | NA | NA | NA |
| Schirrmeister JF et al, 2007 | molecular based | non-surgical main root canal | most common microbiome: E faecalis DNA (31%) was detected by nested PCR (S0). | none of the teeth showed positive cultures. | Microbial load: 12 teeth (60%) contained cultivable microorganisms after removal of the root canal filling material (S0). Of these 12 teeth, the quantity of microorganisms recovered ranged from 0 to 7.8 107 CFU/mL (with a median of 3.5 103 CFU/mL) and from 3.0 103 to 1.0 109 CFU/mL (with a median of 1.5 105 CFU/mL) After irrigation with EDTA and NaOCl (S1) and after irrigation with CHX (S2), none of the teeth showed positive cultures. | NA |
| Zargar N et al, 2019 | culture and molecular based | non-surgical main root canal | Most common microbiome: Enterococcus faecalis 63.63%, Prevotella pallens 6.66%. Periapical lesions less than 5 mm, E. faecalis 80% and Dialister invisus 53.3%. Periapical lesions greater than 5 mm, Dialister invisus, Streptococcus salivarius and Treponema denticola were the most prevalent (53.3%) and E. faecalis (46.7%). Candida albicans have higher prevalence rate in the teeth with PA lesions smaller than 5mm (40%) . HSV 1 more prevalent in teeth with PA lesions smaller than 5 mm (40%) | NA | NA | NA |
| Sánchez‐Sanhueza G et al, 2018 | molecular based | non-surgical main root canal | Most common microbiome: Proteobacteria was the most abundant phyla, followed by Bacteroidetes. In symptomatic patients, a greater abundance of Proteobacteria. In asymptomatic patients, a higher abundance of Bacteroidetes. | NA | NA | NA |
| Siqueira JF et al, 2020 | molecular based | surgical root apices | Most common microbiome: Actinobacteria counts were 2.23 times higher in granulomas than in cysts. Streptococci were significantly more present in small lesion cases. E. faecalis, always as a dominant community member. | NA | NA | CBCT and micro-CT: micro-CT scans of apices of posttreatment apical periodontitis showed that large lesions were significantly associated with a higher volume of unfilled apical canals. |
| Cavrini F et al, 2008 | molecular based | non-surgical - main root canal | most common microbiome: T. denticola detected in 30.3% of teeth with primary infection. and in 34.7% teeth with secondary infection. | NA | NA | NA |
| Pirani C et al, 2008 | molecular based | non-surgical - main root canal | most common microbiome: E. faecalis was detected in (7.6%) with primary lesion, and in (39.1%) with secondary lesion. | NA | NA | NA |
| Teofani A et al, 2024 | molecular based | non-surgical main root canal, mucosa (control samples) | Most common microbiome: Firmicutes is the predominant phylum in the oral controls (34.5%) and PAI-1 (44.4%) groups. Bacteroidetes are predominant in PAI-3 (38.6%). The Proteobacteria (21.5%) and Fusobacteria (12.5%) relative abundance is higher in oral controls while that of Synergistetes is higher in the PAI-1 (3.5%) and PAI-3 (2.5%) groups, being almost absent in controls (less than 0.1%). Most of the increased bacterial species found in the PAI groups were strict anaerobes. | NA | Microbial load: increase in bacteria belonging to phyla and genera with an increased anaerobic character. | NA |
| Vianna ME et al, 2005 | culture and molecular based | non-surgical - main root canal | Most common microbiome found by culture: F. nucleatum (25%), Gemella morbillorum (25%), Eubacterium lentum (20%), and Enterococcus faecalis (20%). Most common microbiome found by the DNA chip : M. micros (50%), F. nucleatum ssp. (40%), T. forsythia (40%), and T. denticola (30%). | NA | NA | NA |
| Siqueira Jr JF et al, 2000 | molecular based | non-surgical - main root canal | Most common microbiome: T. denticola detected in (52.4%) cases, regardless of the presence or absence of symptoms. | NA | NA | NA |
| Zargar N et al, 2020 | molecular based | non-surgical - main root canal | Most common microbiome: Dialister invisus (68.3%), Porphyromonas gingivalis (58.8%), Streptococcus salivarius (58.5%), and Treponema denticola (56.1%). Lysinibacillus fusiformis (19.1%) detected in the root canals for the first time in primary endodontic lesions with the highest prevalence in the necrotic pulp of teeth with PA involvement. Candida albicans detected in (26.8%) with the highest prevalence in the teeth with a necrotic pulp and PA involvement. Herpes simplex virus (HSV) detected in (9.8%) with the highest prevalence in teeth with irreversible pulpitis without PA involvement | NA | NA | NA |
| Lima AR et al, 2021 | molecular based | non-surgical abscess aspiration, main root canal | Most common microbiome: S. mutans showed a high prevalence, except the control group. | NA | NA | NA |
| Rosa TP et al, 2015 | culture and molecular based | surgical root apices and periapical soft lesions | Most common microbiome: Treponema species in 28% of the cases, T. socranskii (24%), followed by T. maltophilum (12%), T. amylovorum (12%), T. lecithinolyticum (12%), T. denticola (12%), T. pectinovorum (8%) and T. medium (8%), T. vicentii was not detected in any sample. using nPCR | NA | NA | NA |
| Qian W et al, 2019 | molecular based | surgical root apices, extraction | Most common microbiota: Streptococcus, Haemophilus (parainfuenzae in particular), Actinomyces, Granulicatella, Leptotrichia. , Firmicutes, Proteroidetes and Bacterioidetes, followed by Actinobacteria, Fusobacteria, Spirochaetes, Synergistetes, Acidobacteria, TM7, Cyanobacteria, Tenericutes, Chloroflexi, SR1, Elusimicrobia, GN02, Planctomycetes, Gemmatimonadetes, [Thermi] and Nitrospirae. | NA | NA | NA |
| Assed S et al, 1996 | molecular based | non-surgical main root canal | Most common microbiota: Actinomyces viscosus (56%), Prevotella intermedia (48%), Fusobacterium nucleatum (40%), Porphyromonas gingivalis (16%). | NA | NA | NA |
| Chávez De Paz LE et al, 2003 | culture , Gas-liquid chromatography | non-surgical main root canal | Most common microbiome: Non-mutans group streptococci, Enterococcus spp., Coagulase negative staphylococci, Peptostreptococcus spp., Mutans group streptococci, Lactobacillus spp. | Most common microbiota: Gram-positives predominated (85%). Lactobacillus spp. (22%), nonmutans streptococci (18%), and Enterococcus spp. (12%) are the most common isolates following root-canal treatment of teeth with apical periodontitis. Gram-negative anaerobes were relatively sporadic. | NA | Periapical radiograph |
| Didilescu AC et al, 2012 | molecular based | non-surgical main root canal | Most common microbiome: In perioendo lesions; In endodontic samples: P. micra, F. nucleatum and C. sputigena. In periodontal samples: the same species, plus C. rectus. | NA | NA | NA |
| Makino K et al, 2015 | molecular based | surgical root apices and periapical soft lesions | Most common microbiome: EBV was detected in 78.1% of samples of periapical granulomas by real-time PCR. | NA | NA | NA |
| Ozbek SM et al, 2016 | molecular based | non-surgical abscess aspiration | Most common microbiome: HCMV 19%, EBV 14%, HPV 5% and HHV-6 DNA 5% in the specimens examined. | NA | NA | Periapical radiograph |
| Siqueira JF Jr & ROCAS IN, 2002 | molecular based | non-surgical main root canal | Most common microbiome: Dialister pneumosintes detected in primary root canal infections (65.6%). D pneumosintes detected in root canals associated with chronic asymptomatic periradicular lesions (77.3%). associated with acute apical periodontitis (40%). | NA | NA | NA |
| Abushouk S et al, 2021 | molecular based | non-surgical main root canal | Most common microbiome: F. nucleatum (57.3%), T. denticola (42.7%), P. endodontalis (29.3%) in apical periodontitis associated with caries. P. endodontalis at high detection threshold is associated with swelling. | NA | NA | NA |
| Andric M et al, 2007 | molecular based | surgical periapical soft lesions | Most common microbiome: Cytomegalo Virus (CMV) detected in Periapical Cysts (54.5%) and Odontogenic Keratocysts (60%). | NA | NA | NA |
| Barani M et al, 2023 | culture based | non-surgical main root canal | Most common microbiome: Streptococcus mitis (14.8%)- Streptococcus oralis (9.8%) Granulicatella elegans (9.8%)- Enterococcus faecalis (4.9%)- Clostridium bifermentans (13.5%) | Acute pulpitis: Anaerobic: Clostridium bifermentans, Clostridium barotii, Aerobic: Streptococcus mitis, Granulicatella elegans Chronic pulpitis: Anaerobic: Clostridium barotii, Clostridium bifermentans, Aerobic: Streptococcus oralis, Streptococcus mitis. | NA | NA |
| Garcez AS et al, 2015 | Culture Based Methods. | surgical periapical lesion | NA | NA | Microbial reduction: Combined surgical and PDT treatment achieved 5-log reduction; surgery alone achieved 3.5-log reduction; surgery alone achieved 3.5-log reduction. | Periapical radiograph: Average lesion size reduction: 78%. |
| Gomes BPFA et al, 2007 | molecular based | non-surgical main root canal | Most common microbiome: Porphyromonas gingivalis (44%)- Treponema denticola ( 38%)- Tannerella forsythia ( 24%)- “Red complex” (all three species) in cases (14%) | NA | NA | NA |
| Sassone L et al, 2007 | molecular based | non-surgical main root canal | Most common microbiome: - Enterococcus faecalis (89.3%)- Campylobacter gracilis (89.3%)- Leptotrichia buccalis (89.3%)-Neisseria mucosa (87.5%)- Prevotella melaninogenica (86.6%)- Fusobacterium nucleatum ssp. vincentii (85.7%)- | NA | Microbial load: Highest mean counts (>10⁵): - F. nucleatum ssp. vincentii (13.14 × 10⁵)- Eubacterium saburreum (5.67 × 10⁵)- E. faecalis (5.38 × 10⁵). | NA |
| Gomes BPFA et al, 2006 | culture and molecular based | non-surgical main root canal | Most common microbiome: Enterococcus faecalis Detected by PCR in 82% of primary infections and 76% of secondary infections- Cultured in 4% of primary infections and 42% of secondary infections. | NA | NA | NA |
| Cao H et al, 2012 | molecular based | non-surgical main root canal | Most common microbiome: Porphyromonas endodontalis (50%)- Prevotella intermedia (45%)- Porphyromonas gingivalis (33%). | NA | NA | NA |
| Stojanović N et al, 2014 | molecular based | non-surgical main root canal | Most common microbiome: Enterococcus faecalis: 49% - Porphyromonas gingivalis: 17.6% | E. faecalis in 3.4% (1/29), P. gingivalis in 0% after 15-day medication (S3) | Microbial reduction: Significant microbial reduction observed: - From S1 to S2 (p<0.001)- From S2 to S3 (p<0.05)- From S1 to S3 (p<0.001) Different intracanal medications showed similar reduction trends. | NA |
| Gomes BPFA et al, 2005 | culture and molecular based | non-surgical main root canal | Most common microbiome: Porphyromonas gingivalis (38%), Prevotella intermedia (33%), Porphyromonas endodontalis (25%), Prevotella nigrescens (22%). Additional species detected by culture: Prevotella corporis, P. loescheii, P. denticola, P. melaninogenica | NA | NA | NA |
| Murad CF et al, 2014 | molecular based | non-surgical main root canal | Most common microbiome: Enterococcus faecium (37%) Staphylococcus epidermidis (37%) Eubacterium saburreum (28%) Parvimonas micra (28%) Streptococcus sanguis (28%)Capnocytophaga sputigena (28%)Leptotrichia buccalis (28%) Enterococcus faecalis (28%) Staphylococcus warneri (28%) | NA | Microbial load: Gram-negative species: 13.5 × 10⁵ Gram-positive species: 6.5 × 10⁵ Helicobacter pylori had high individual levels (0.86 × 10⁵) despite low prevalence (10%). | NA |
| Foschi F et al, 2005 | molecular based | non-surgical main root canal | Most common micrbiome: T. denticola and E. faecalis (24%), P. gingivalis (13%), P. intermedia (8%), and T. forsythensis (7%). T. denticola was detected in 56% of teeth with EAP. E. faecalis was found in 60% of teeth with CAP and in 72% of teeth with secondary infection. | NA | NA | NA |
| Hu Z et al, 2023 | molecular based | non-surgical main root canal | Most common microbiome: Porphyromonas gingivalis (most abundant overall), Fusobacterium nucleatum (detected in all samples; most variable), Pyramidobacter piscolens, Dialister invisus, Porphyromonas endodontalis, Parvimonas micra, Filifactor alocis, Prevotella oris, Phocaeicola abscessus | NA | NA | NA |
| Zeledón R et al, 2015 | molecular based | non-surgical main root canal | Most common microbiome: Actinomyces Israelii > Enterococcus Faecalis > Fusobacterium Nucleatum/ Prevotella Nigrescens > Phorphyromonas Endodontalis. | Prevalence of Actinomyces Israelii (16%), Enterococcus Faecalis (14%) and Phorphyromonas Endodontalis in final samples S4 | NA | Periapical radiograph: the correlation of PCR results with the radiological follow-up demonstrates that in the six cases tabulated as unsuccessful (PAI 3, 4 and 5) were detectable the persistence of Actinomyces israelii and Enterococcus faecalis after Intracanal medication or before obturation |
| Gomes BP et al, 2021 | Culture and molecular based | non-surgical main root canal | Most common microbiome detected by molecular method: molecular analyses; P. gingivalis, E. faecalis, and Fusobacterium nucleatum. Most frequently identified genera: Staphylococcus (40%), Gemella (38%), Actinomyces (36%), Enterococcus (30%), Streptococcus (26%), and Aerococcus (26%).  Most common microbiome detected using culture method: Of the 63 species detected by culture, the most frequently identified species were E. faecalis (28%), G. morbillorum (28%), Aerococcus viridans (26%), Gemella haemolysans (16%), and Staphylococcus lentus (16%) | NA | NA | NA |
| Peters LB and Wesselink PR, 2002 | Culture Based | non-surgical main root canal | NA | NA | Microbial load: Prior to the canal obturation, there were eight teeth (seven in group 1, one in group 2) with a positive root canal culture. Six positive root canals contained <10^2 CFU mL^-1, one canal contained 2 x 10^3 and one canal harboured 8 x10^4 CFU mL^-1. Of the 30 cases that were filled with a negative culture prior to obturation, 22 (74%) healed (11 from group 1 and 11 from group 2) whereas this was the case for seven out of eight cases (six from group 1 and one from group 2) with a positive root-canal culture prior to obturation (87.5%). No significant difference in healing of periapical radiolucency was observed between teeth that were treated in one visit (without) and two visits with inclusion of calcium hydroxide for 4 weeks. The presence of a positive bacterial culture (CFU < 10^2) at the time of filling did not influence the outcome of treatment . | Periapical radiograph: At the end of the follow-up period 18% still had a radiolucency (periapical score 4 and 5), in 82% a clear radiolucency was not present (periapical score 1 and 2). The periapical scores after the follow-up period were significantly lower. |
| Moghadam MD et al, 2021 | culture based | non-surgical main root canal | NA | NA | Microbial load: At S1, no significant difference was noted among the three groups in microbial load. Microbial reduction: after root canal preparation S2 and One week after intra canal medicament S3, there was a statistically significant lower bacterial count in Diode Laser (P=0.041) and depotphoresis group (P=0.001) respectively compared to other groups | CBCT: at 6 months follow up; no significant difference in healing status of the lesions with mesiodistal and buccolingual diameter ≤5 mm and >5 mm in depotphoresis group (P>0.05). However, this difference was significant between lesions with apicocoronal diameter ≤5 mm and >5 mm (P=0.037) such that 33.33% of such lesions had completely healed. No significant difference was noted in healing status of the lesions with diameter ≤5 mm and >5 mm in the laser group (P>0.05). Also, none of the lesions in the control group showed complete healing. Except in depotphoresis group that the success rate was higher in the cases with lesions ≤5 mm in apicocoronal diameter (P=0.042), there was no positive correlation between the outcome of the therapy and the size of the periapical lesions diameters in all groups (p>0.05). |
| Nogales CG et al, 2025 | molecular based | non-surgical main root canal | NA | NA | Microbial reduction: DNA-based methods revealed a significant decrease in bacterial levels from S1 to S2 and S2 to S3 . Notably, 11 out of 35 (31.4%) root canals did not harbor bacterial DNA after CMP, whereas ultrasonic activation increased DNA-negative samples to 17 (48.6%). | NA |
| Sedgley C et al, 2006 | Culture and molecular based | non-surgical main root canal | Most common microbiome: Using qPCR, E. faecalis was detected in 79.5% of total samples. Detected in 67.5% of primary infections Detected in 89.6% of retreatment cases. Culture methods detected E. faecalis in only 10.2% of cases. Other bacteria (from culture): Streptococcus sanguis, Proteus spp., non-polysaccharide producing Streptococcus spp., and gram-positive anaerobic rods . | NA | Microbial load: qPCR detected total bacterial loads ranging from 5.2 × 10³ to 1.4 × 10⁷ cells/100 µL (mean: 2.0 × 10⁶). In E. faecalis-positive samples, counts ranged from 1.1 × 10³ to 1.7 × 10⁶ cells/100 µL (mean: 8.7 × 10⁴). Culture was significantly less sensitive; the most positive culture results showed <10⁴ CFU per 100 µL. qPCR showed significantly higher detection of both E. faecalis and total bacteria compared to culture. | NA |
| Siqueira JF Jr et al, 2016 | molecular based | surgical root apices | Most common microbiomes: Phyla; Proteobacteria (46%), Firmicutes (18%), Fusobacteria (15%), Actinobacteria (8%). Genera; Fusobacterium (15%), Pseudomonas (15%). P | NA | NA | NA |
| Gomes BPFA et al, 2015 | culture and molecular based | non-surgical main root canal, periodontal pockets | Most common microbiomes: Enterococcus faecalis, Parvimonas micra, Mogibacterium timidum, Filifactor alocis, and Fretibacterium fastidiosum. | Enterococcus faecalis, Parvimonas micra, Mogibacterium timidum, Filifactor alocis, and Fretibacterium fastidiosum. | NA | NA |
| Zakaria MN et al, 2015 | molecular based | surgical, root apices | Most common microbiome: Porphyromonas gingivalis (19.8%), Propionibacterium acne (17.2%), Fusobacterium nucleatum (11.3%), Streptococcus mitis (4.2%), Peptostreptococcaceae sp. HOT-113 (4.2%) | NA | Microbial load: Total bacteria per sample: 5.1×10³ to 1.9×10⁶ cells (mean: 2.0×10⁵ ± 5.2×10⁵), Bacterial load higher in symptomatic vs. asymptomatic lesions (3.3×10⁵ vs. 1.1×10⁵ cells) | NA |
| Sanghavi TH et al, 2014 | molecular based | non-surgical main root canal | Most common microbiome: Treponema denticola, Porphyromonas gingivalis, Tannerella forsythia | NA | NA | NA |
| Blome B et al, 2008 | molecular based | non-surgical main root canal | Most common microbiome: Peptostreptococcus micros (55%) P. endodontalis (35%) T. denticola (22.5%) T. forsythia (30%) F. nucleatum (27.5%) | NA | Microbial reduction: Primary infection group (untreated teeth): Initial bacterial count: 1.5 × 10^7 After root canal preparation: 2.3 × 10^4 (a 99.9% reduction) After calcium hydroxide dressing: 8.6 × 10^3 Secondary infection group (root-filled teeth): Initial bacterial count: 2.6 × 10^5 After root canal preparation: 6.4 × 10^3 (a 97.3% reduction) After calcium hydroxide dressing: 7.1 × 10^3 | NA |
| Nobrega LM et al, 2016 | molecular based | non-surgical main root canal | Most common microbiome: Prevotella spp. (e.g., Prevotella oris), Fusobacterium nucleatum, Filifactor alocis ,Peptostreptococcus stomatis ,Dialister invisus, Phocaeicola abscessus | NA | NA | NA |
| Arias‐Moliz MT et al, 2024 | molecular based | surgical root apices, periapical lesions | Most common microbiome: Fusobacterium nucleatum (19.8%) Prevotella loescheii (4.18%) Streptococcus intermedius (4.17%) Porphyromonas gingivalis (4.06%) Parvimonas micra (3.9%) Synergistetes bacterium (3.37%) | Fusobacterium nucleatum (19.8%) Prevotella loescheii (4.18%) Streptococcus intermedius (4.17%) Porphyromonas gingivalis (4.06%) Parvimonas micra (3.9%) Synergistetes bacterium (3.37%) | NA | NA |
| Henriques LC et al, 2016 | molecular based | non-surgical main root canal | Most common microbiome: Corynebacterium diphtheriae • Porphyromonas gingivalis • Streptococcus sobrinus • Stenotrophomonas maltophilia | NA | NA | NA |
| Sunde PT et al, 2001 | molecular based | surgical periapical lesion | Most common microbiome: Group 1 (Marginal incision, more contaminated): Streptococcus gordonii,Selenomonas noxia,Prevotella intermedia,Capnocytophaga gingivalis,Fusobacterium nucleatum ssp. polymorphum,Treponema denticola Group 2 (Submarginal incision): Fusobacterium nucleatum ssp. vincentii,Veillonella parvula,Streptococcus anginosus,Streptococcus gordonii,Peptostreptococcus micros,Actinomyces israelii | NA | Microbial load: High bacterial counts (≥10⁶ cells) were reported for several species, especially in Group 1 | NA |
| Ribeiro AC et al, 2011 | molecular based | non-surgical main root canal | Most common microbiome: Atopobium rimae (50.0%) Dialister invisus (33.3%) Prevotella oris (33.3%) Pseudoramibacter alactolyticus (33.3%) Tannerella forsythia (33.3%) | NA | NA | NA |
| Jacinto RC et al, 2008 | Culture based | non-surgical main root canal | Most common microbiome: Fusobacterium nucleatum (34.5%) , Fusobacterium necrophorum (18.2%) isolated from root canals | NA | Microbial load: 580 isolates from 110 root canals; Up to 9 species per canal, 81.4% (472/580) were strict anaerobes,45% (261/580) were gram-negative bacteria | NA |
| Sassone LM et al, 2008 | molecular based | non-surgical main root canal | Most common microbiome: Fusobacterium nucleatum ssp. vincentii , Veillonella parvula, Treponema socranskii , Enterococcus faecalis, Campylobacter gracilis . | NA | Microbial load: Fusobacterium nucleatum ssp. vincentii (15.38 ± 3.38 × 10⁵), Veillonella parvula (7.87 ± 2.51 × 10⁵), Treponema socranskii (7.02 ± 2.53 × 10⁵), Enterococcus faecalis (6.62 ± 1.80 × 10⁵), Campylobacter gracilis (5.55 ± 1.80 × 10⁵). The total levels of bacteria observed in symptomatic cases was 86 × 10⁵ (SE = 24 × 10⁵), and 29 × 10⁵ (SE = 8.4 × 10⁵) in asymptomatic cases. | NA |
| Hou Y et al, 2021 | molecular based | non-surgical main root canal | Most common microbiome: Genera; Porphyromonas (6.82%), Fusobacterium (5.96%), Faecalibacterium (5.70%), Prevotella (4.22%), Phocaeicola (3.53%), Streptococcus (3.31%), Parvimonas (2.95%). The most abundant genera in the SAP group apical segments; Fusobacterium (13.32%), Porphyromonas (11.67%), Phocaeicola (8.76%), Streptococcus (5.28%), Faecalibacterium (3.56%) | NA | NA | NA |
| Sabeti M and Slots J, 2004 | Culture based, viral detection | surgical periapical lesion | Most common microbiomes: Streptococcus, Fusobacterium, P. micros, Staphylococcus, Campylobacter | NA | NA | NA |
| Geibel M et al, 2005 | molecular based | surgical periapical lesion | Most common microbiome: Micromonas micros (gram positive coccus), F. nucleatum, E. faecalis, S. sanguinis, E. coli or P. aeruginosa | NA | NA | NA |
| Cardoso FG et al, 2016 | Culture; and PCR | non-surgical main root canal | Most common microbiome: Porphyromonas endodontalis | NA | NA | NA |
| Tiwari S et al, 2020 | molecular based | non-surgical main root canal | Most common microbiome: P. gingivalis most prevalent, followed by T. forsythia and T. denticola | NA | NA | NA |
| Bogen G and Slots J, 1999 | Culture and molecular based | surgical periapical lesion | NA | NA | NA | NA |
| Neves MA et al, 2020 | molecular based | non-surgical main root canal | NA | NA | Microbial reduction: Quantitative bacterial reduction was similar between groups (p>0.05). A difference of >1 Log10 counts was observed between healed and diseased cases. | Periapical radiograph: Mean for the follow-up period was 20.5 months for BioRaCe and 17.4 months for Reciproc. The mean (median) PAI score decreased from 4.1 4 to 1.8 1 in the Bio Race group, and from 3.7 4 to 2 2 in the Reciproc group. |
| Mussano F et al, 2018 | molecular based | surgical periapical lesion | Most common microbiome: Lactococcus lactis, Propionibacterium acnes, Staphylococcus warneri, Acinetobacter johnsonii and Gemellales. L. lactis (facultative anaerobes taxa) | NA | NA | NA |
| de Azevedo Moreira S 2021 | molecular based | non-surgical main root canal | Most common microbiome: Enterococcus faecalis | most common microbiome: Actinomyces israelii | microbial load: Evaluation of the two bacteria between collections 1, 2 and 3, showed that there was no difference | Periapical radiograph: There was association between the variables group and repair classification in radiographs evaluation |
| Machado et al, 2020 | culture technique and molecular based | non-surgical intra canal | Most common microbiome: in 1ry AP; The most prevalent gram-positive bacterial species (S. intermedius (45.16%), E. faecalis (41.94%), P. acnes (41.94%), and S. constellatus (35.48%). The most prevalent gram-negative bacterial species (P. gingivalis (–45.16%), C. sputigena (41.94%), P. melaninogenica (41.94%), and L. buccalis (38.71%)). most common microbiome in 2ry AP; The most prevalent gram-positive bacterial species (E.nodatum (65.62%), S. mitis (62.07%), E. faecalis, S. constellatus, E. faecium, and E. saburreum (58.62%)). The most prevalent gram-negative bacterial species (P. gingivalis (72.41%), C. rectus (68.97%), C. sputigena, and F. nucleatum. sp. vicentii (65.62%). | NA | NA | CBCT analysis: The median volume of bone destruction determined by CBCT analysis in 1ry and 2ry was 60 mm3 (20–280 mm3) and 60 mm3 (10–680 mm3) Although the CBCT data showed similarity in both infections, it is worth noting that despite the absence of statistical difference 2ry presented lesion volume values higher than 1ry." |
| Ahmed S et al 2024 | Culture-Based Methods. | non-surgical main root canal | Most common microbiome: E. faecalis and C. albicans | NA | NA | NA |
| Kist S et al, 2017 | molecular based | non-surgical main root canal | Most common microbiome: facultative anaerobe Gram-positive cocci Streptococcus spp, Gram-positive anaerobe cocci Parvimonas spp, and the obligate anaerobic Gram-negative rods Prevotella spp. | NA | Microbial reduction: The reduction rates (%) between the initial bacterial load and the number of CFU ml−1 after chemo-mechanical treatment (sample 2) and after inter-appointment dressing (sample 3) in the ozone group and the NaOCl group were very similar. | Periapical radiograph: decrease in PAI values and apical lesion sizes were also insignificant after 6 and 12 months |
| Egan MW et al, 2002 | Molecular Based | non-surgical main root canal | Most common microbiome: Candida albicans (73.9% & 37.5%) and Rodotorula mucilaginosa (8.6%& 50%) were the most prevalent isolates from saliva and root canal samples | NA | NA | NA |
| Vianna ME et al, 2007 | molecular based | non-surgical main root canal | Most common microbiome: Treponema (56.25%), Porphyromonas gingivalis (28.125%), Prevotella intermedia (15.6%), | NA | NA | NA |
| Siqueira JF and Rocas IN, 2003 | molecular based | non-surgical main root canal | most common microbiome: Campylobacter gracilis and C. rectus | NA | NA | NA |
| Donnermeyer D et al, 2025 | molecular based | non-surgical main root canal | Most common microbiome: Pseudomonadales, which included the genera Acinetobacter, Moraxella, and Pseudomonas | most common microbiome: Fusobacterium and Prevotella | NA | NA |
| Niazi SA et al, 2016 | molecular based | non-surgical main root canal | Most common microbiome: Propionibacterium acnes as the most prevalent from lesions with communication.  Microbiota of endodontic infections without communication with the oral cavity: Actinomyces naeslundii (57 %) was the most prevalent bacterial taxa, followed by Streptococcus gordonii, Streptococcus mitis bv, Streptococcus sanguinis, V. dispar and V. parvula (28.57 %). Microbiota of Primary endodontic infection with “open” communications with the oral cavity: Propionibacterium acnes the most prevalent bacterial taxa. Staphylococcus epidermidis was also recovered. | NA | NA | NA |
| Ahlat M et al, 2023 | molecular based | non-surgical main root canal | Most common microbiome: (Bacillota, Bacteroidota, Pseudomonadota, Fusobacteriota and Ascomycota). The most microorganism species identified were Enterococcus faecalis Lactobacillus rhamnosus.  Lactobacillus paracasei, Enterococcus faecalis | NA | NA | NA |
| Alquria TA et al, 2024 | molecular based | non-surgical main root canal | Most common microbiomes: Bacteroidetes (38.37%), Firmicutes (19.58%), and Synergistetes (7.44%). | Most common microbiomes: Firmicutes (30.77%), Bacteroidetes (24.11%), and Actinobacteria (8.04%) after CMP s2. The CMP with 2.5% NaOCl showed a significant impact on the bacteriome composition. | Microbial load: The qPCR counts were significantly higher in s1 than s2. Microbial diversity: AChao1 index indicated no difference in alpha diversity, whereas Shannon and Simpson indexes showed higher values in s2 | NA |
| Blome B et al, 2008 | molecular based | non-surgical main root canal | Most common microbiome: P. micros (55%) and P. endodontalis(35%) were the most frequently detected species in both primary and secondary | NA | Microbial reduction: the use of calcium hydroxide as an intracanal dressing for 14 days did not lead to a further reduction of the total bacterial counts; the detection frequency of individual bacterial species was further reduced | NA |
| Hommez GM et al, 2004 | molecular based | non-surgical main root canal | Most common microbiome: Fusobacterium nucleatum/Streptococcus mitis, Veillonella sp. | NA | NA | NA |
| Cardoso FG et al, 2015 | Culture Based Method. | non-surgical main root canal | NA | NA | microbial load: Bacteria and endotoxins were detected in 100% of the root canal samples. with median values of 7.5x 10^5 CFU/mL (3.20 x10^5–8.16 x 10^6 CFU/mL) and 10.92 EU/mL (1.75–128 EU/mL) | NA |
| Moraes SR et al, 2002 | culture and molecular based | non-surgical main root canal | NA | NA | NA | NA |
| Pelozo LL et al, 2023 | culture Based Method | non-surgical main root canal | NA | NA | Microbial reduction: in S2, the laser provided 42.44% microbial reduction and 53.14% of E. faecalis, different from the placebo that had no reduction, and 4.85% for Enterococcus (P < 0.05). In S3, the bacterial counts decreased without differences between groups | Periapical radiograph: After 1 year, the recall rate was 83.3% and the complete healing rates (score 1) were significantly different (P < 0.05) between the laser (62%) and the placebo group (17%) |
| Vianna ME et al, 2006 | molecular based | non-surgical main root canal | Most common microbiome: positive for methanogens (25%), the archaeal community was dominated by a Methanobrevibacter oralis-like phylotype | NA | NA | NA |
| Gomes BP et al, 2020 | molecular based. | non-surgical main root canal | Most common microbiome: F. alocis DNA was detected in 46% RCs of teeth with PEI and in 24%RCs of teeth with SEI. | NA | Microbial load: Microbial load of F. alocis was higher in PEI than in SEI | NA |
| Carneiro E et al, 2017 | Immunohistochemistry and gram stain | surgical the periapical lesion | NA | NA | NA | NA |
| Martinho FC et al, 2010 | molecular based | non-surgical main root canal | Most common microbiome: Prevotella nigrescens (57.2 %) was the most frequent species | NA | NA | NA |
| Garcez AS et al, 2008 | culture based | non-surgical main root canal | NA | NA | Microbial reduction: The surgical procedure alone achieved a mean ~3.5-log reduction in bacterial load, whereas adjunctive aPDT achieved an additional ~5-log reduction, | NA |
| Sabeti M et al, 2003 | molecular based | surgical root apices | Most common microbiome: HCMV and EBV, HCMV transcript was detected in 92.3% symptomatic and in 7.69% asymptomatic periapical lesion. EBV transcript was demonstrated in 61.53% symptomatic lesions but not in the asymptomatic periapical lesion. | NA | NA | NA |
| Tawfik SA et al, 2018 | molecular based | non-surgical intra canal | Most common microbiome: At phyla level; Firmicutes, Bacteroidetes, Proteobacteria, and Synergistetes. At genus level; Prevotella, Bacillus, Porphyromonas, Streptococcus,andBacteroides . | NA | NA | NA |
| Siqueira Jr JF and Rôças IN, 2017 | molecular based | non-surgical aspiration | Most common microbiome: Fusobacterium nucleatum, Parvimonas micra, Porphyromonas endodontalis, Olsenella uli, streptococci, Eikenella corrodens, some as-yet-uncultivated phylotypes (Bacteroidetes clone X083 and Synergistes clone BA121), and newly named species (Prevotella baroniae and Dialister invisus) | NA | NA | NA |
| Pereira R et al,2017 | molecular based | surgical root apices, periapical lesions | Most common microbiome: Fusobacterium nucleatum (71.6%), Dialister pneumosintes (58.3%) and Tannerella forsythia (48.3%) in both root end and periapical samples. Dialister pneumosintes showed statistically significant values in the root end, and F. nucleatum was also significant in the apical periodontitis samples | NA | NA | NA |
| Carvalho AP et al, 2020 | molecular based | non-surgical intra canal | NA | NA | Microbial reduction: a significant reduction in the median number of bacterial cells in the S2 samples, compared to S1. No significant decrease in bacterial counts was found when comparing S2 with S3a, S3b, or S4. However, the bacterial counts in S5 were significantly lower than in S2 . In the qualitative (presence/absence) analysis, significant differences in the number of positive cases were observed between S1 and S2 and S4 and S5 . | NA |
| Cavalli D et al, 2017 | culture and molecular based | non-surgical intra canal | NA | Most common microbiome: F. nucleatum ssp vicentii(50%), L. buccalis (50%), and P. gingivalis (46.70%) at S2. | NA | NA |
| Card SJ et al, 2002 | Culture method and PMR method | non-surgical intra canal | NA | NA | Microbial reduction: 100% of the cuspid/bicuspid canals and 81.5% of the molar canals were rendered bacteria-free after S1 . The molar results improved to 89% after the S2. Of the (59.3%) molar mesial canals without clinically detectable communication, 93% were rendered bacteria-free with the S1. The differences between the samples that followed S1 and S2 were not significant. | NA |
| de Miranda RG and Colombo AP, 2018 | molecular based | non-surgical intra canal | Most common microbiome: Candida albicans (46.9%), Dialister pneumosintes (31.2%), Prevotella nigrescens (28.2%), Prevotella tannerae (28.1%), and Peptostreptococcus anaerobius (25%). | Most common microbiomes: C. albicans and D. pneumosintes were still detected in high frequency in both groups at 3 months post-therapy. | NA | Periapical radiograph: Significant decreases in PAI scores were observed in both groups over time, although at 6 months, the PDT group presented a significantly better healing score than the control. |
| Buonavoglia A et al, 2023 | molecular based | non-surgical intra canal | Most common microbiome: Firmicutes (27.9%), Bacteroidetes (25.8%), Actinobacteria (17.7%) at the phylum level. Phocaeicola (8.6%), Pseudomonas (7.7%), Rothia (7.5%), and Prevotella (6.8%) the most prominent genera. In samples of patients with AP symptoms, the most frequent genera were Cutibacterium, Lactobacillus, Pseudomonas, Dialister, Prevotella, and Staphylococcus. In PAP samples, were Cutibacterium, Lactobacillus, Pseudomonas, and Prevotella. In SAP cases were Cutibacterium, Prevotella, Atopobium, Capnocytophaga, Fusobacterium, Pseudomonas, Solobacterium, and Streptococcus. | NA | NA | NA |
| Bronzato JD et al, 2021 | molecular based | surgical root apices | Most common microbiome: Parvimonas micra, followed by Enterococcus faecalis, Fusobacterium nucleatum and Porphyromonas endodontalis. | NA | Microbial diversity: The type of endodontic treatment, whether 1ry or 2ry, was not associated with the presence of any bacterial species in periapical lesions. | NA |
| Waltimo T et al, 2005 | culture and molecular based | non-surgical intra canal | NA | At first appointment: showed generally mixed infections dominated by anaerobic microorganisms. At second appointment for Calcium Hydroxide group and Empty Canal group showed Gram-positive facultative microorganisms slightly dominated. Sodium hypochlorite was effective also at the second appointment and only two teeth remained culture positive | Microbial load: The access sample in the first appointment showed bacterial growth in Single visit, Calcium Hydroxide and Empty Canal groups with growth percentages of 20, 22, and 33%, respectively. In the second appointment, Sodium hypochlorite was effective at the second appointment, no growth was detected in CH group and only two teeth (17%) remained culture positive in EC group. | Periapical radiograph: In the Bacteria Absent group the mean change of PAI at the 1-yr control was 1.53, whereas the Bacteria Present group showed only 0.79 mean change of PAI. This difference was statistically significant |
| Noguchi et al | molecular based | surgical root apices | Most common microbiome: Fusobacterium nucleatum (100%), Porphyromo nas gingivalis (86%), and Tannellera forsythensis (57%). | NA | NA | NA |
| Kesim B et al, 2023 | molecular based | non-surgical intra canal | Most common microbiome: In the PEI, Within the Gram-negative facultative anaerobic Gamma proteobacteria class outgroup, two orders (Pasteurellales, Vibrionales) and two families (Pasteurellaceae, Vibrionaceae) , whereas Gram-positive bacteria, Actinomycetales order, and Gram-positive anaerobic taxa, one genus (Olsenella) and one species (Olsenella uli), were identifed as signifcantly more abundant in the SEI. | NA | NA | NA |
| Gajan EB et al, 2009 | Culture Based Method. | non-surgical intra canal | Most common microbiome: Peptostreptococcus, Streptococcus, Porphyromonas, and Enterococcus faecalis. Strains of P. provetti, S. sanguis, S. salivarius, P. endodontalis, and especially E. faecalis were prevalent in the 2ry AP . C. albicans, Veillonella spp., E. coli, Actinomyces meteri, Fusobacterium, Eubacterium lertum, and S. oralis were only found in 1ry Ap. S. salivarius, P. endodontalis, A. odontolyticus, and Peptostreptococcus provetti were almost equally found in both 1ry and 2ry AP. | NA | NA | NA |
| Sabeti M et al, 2003 | molecular based. | surgical periapical lesion | Most common microbiome: Active HCMV and EBV | NA | NA | NA |
| Sabeti M et al, 2003 | molecular based | surgical periapical lesion | Most common microbiome: HCMV and EBV were shown in transcription of the lesion. | NA | NA | NA |
| Hepsenoglu YE and Ersahan S, 2023 | molecular based | non-surgical main root canal | Most common microbiome: Enterococcus (35%), Streptococcus (25%) , Lactobacillus (10%) , Fusobacterium (10%) i, Bacillus (5%) , Neisseria (5%) in , Alkalibacterium (5%), and Pseudoramibacter (5%). | Most common microbiome: Fusobacterium nucleatum, Lactobacillus species, Enterococcus species, Streptococcus intermedius | Microbial reduction: Subsequent activation resulted in a significant microbial reduction in both XPF and EA groups. both of which reduced significantly more bacteria than chemomechanical instrumentation with no statistical difference between the XPF group and the EA group in terms of E. faecalis copy number | NA |
| Xavier A et al,2014 | culture and molecular based | non-surgical main root canal | Most common microbiome: Enterococcus faecalis strains | NA | NA | NA |
| Barbosa-Ribeiro M et al, 2020 | culture and molecular based | non-surgical main root canal | Most common microbiome: E. faecalis (100) %, P. gingivalis (100%), F. nucleatum (85%) and A. actinomycetemcomitans (50%). | A. israelii, A. naeslundii, G. morbillorum, T. forsythia and T. denticola were not detected and F. nucleatum had its DNA significantly reduced after CMP | NA | NA |
| Abou-rass M and Bogen G,1998 | culture based | surgical, root apices | Most common microbiome: Actinomyces sp. (31.8%), Propionibacterium sp. (22.7%), Streptococcus sp (18.2%), Staphlyococcus sp. (13.6%), Porphyromonas gingivalis (4.6%), Peptostreptococcus micros (4.6% ) and Gram-negative enterics ( 4.6% ). | NA | NA | NA |
| Pinheiro E et al,2002 | Culture method (advanced microbiological techniques for anaerobic species) | non-surgical main root canal | Most common microbiome: Facultative anaerobic species (57.4%) and Gram-positive microorganisms (83.3%). Enterococcus faecalis was the most frequently recovered bacterial species. Obligate anaerobes accounted for 42.6% of the species. Peptostreptococcus was the most frequently isolated genera associated with clinical symptoms | NA | NA | NA |
| Ozbek S et al,2008 | culture method and Real-time PCR SYBR Green | non-surgical main root canal | Most common microbiome: E. faecalis was detected in (51.9%) patients, suggesting that it exists in not less than 61% of all endodontic infections. | NA | NA | NA |
| Martinho F et al,2014 | molecular based | non-surgical main root canal | Most common micrbiome: Prevotella nigrescens (57.2 %). | NA | Microbial load: increased the number of Gram-negative bacterial species for the presence of a larger area of bone destruction, | NA |
| Pattanshetty S et al,2017 | molecular based | non-surgical main root canal | Most common microbiome: Treponema denticola (T. denticola) (42%) in SAP and (58%) in AAP | NA | Micrbial load: The mean counts of T. denticola and F. nucleatum were significantly high in asymptomatic apical periodontitis. For symptomatic apical periodontitis, P. gingivalis, T. denticola, and F. nucleatum | NA |
| Sousa E et al,2012 | culture based | non-surgical main root canal | Most common microbiome: A. prevotii, P. micra, and F. necrophorum. Gram-positive anaerobic bacteria predominated in the root canals with periapical abscesses | NA | NA | NA |
| Ersahan S et al,2022 | molecular based | non-surgical main root canal | Most common microbiome: Enterococcus (37.5%), Streptococcus(25%), Enterobacter (18.7%), Eubacterium(6.2%),Lactobacillus (6.2%) and  Bacillus (6.2%). | NA | NA | Periapical radiograph: The Periapical Index (PAI) score of the CHX group was found to be lower compared with the CH group (p < 0.05) |
| Al-Samahi S and A. Al-Omari M,2014 | molecular based | non-surgical main root canal | Most common microbiome: Actinobacteria (57.0%), Firmicute (27.4%), Proteobacteria (12.3%) and Bacteroides (3.4%) | NA | Microbial load: 179 bacterial species. Actinomyces viscosus and Streptococcus sanguis were associated with the presence of pain. While Staphylococcus haemolyticus and Veillonella spp. were associated with pain of palpation, the pain to percussion was associated with Actinomyces spp., Actinomyces naeslundii, and Actinomyces viscosus. Also, Streptococcus mitis, Bacteroides spp., and Veillonella spp. were associated with periapical swelling. The presence of sinus tract was found to be significantly associated with Neisseria spp. and Staphylococcus haemolyticus. The wet canal was significantly associated with the presence of Propionibacterium acnes and Bacteroides spp. | NA |
| Heling I et al,2001 | molecular based | non-surgical main root canal and surgical, root apices | Most common microbiome: Herpes simplex virus DNA was not detected in the tissue and the saliva samples. | NP | NA | NA |
| Tzanetakis G et al,2015 | molecular based | non-surgical main root canal | Most common microbiome: Bacteroidetes in primary and persistent infections. Proteobacteria and Tenericutes in persistent infections; including increased enrichment of persistent infections for Lactobacillus, Streptococcus, and Sphingomonas at genus level | NA | NA | NA |
| Kaufman B et al,2005 | molecular based | non-surgical main root canal | Most common microbiome: was Enterococcus spp. | NA | NA | NA |
| Tanumihardja M et al,2014 | cultural based | non-surgical main root canal | Most Common microbiome: Porphyromonas spp in symptomatic apical periodontitis. Streptococcus spp and Porphyromonasspp in asymptomatic apical periodontitis. | NA | NA | NA |
| Zhang S et al, 2010 | molecular based | surgical periapical lesion | Most common microbiome:Porphy- romonas endodontalis (45% of sample), Actinomyces viscosus (42%), Candida albicans (36%) and Porphyromonas gingivalis (27%). Fusobacterium (27%), Actinomyces israelii (21%) and Enterococcus faecalis (15%) were also detected. | NA | NA | NA |
| Brenda P. F. A et al, 2021 | culture and molecular based | non-surgical main root canal | Most common microbiom: Enterococcus faecalis and Gemella morbillorum, whereas molecular analyses showed a high frequency of P. gingivalis, E. faecalis, and Fusobacterium nucleatum. | NA | NA | NA |
| Pinheiro E.T. et al,2003 | cultural based | non-surgical main root canal | Most common microbiom: Enterococcus (36.7%), Streptococcus (30%), Peptostreptococcus (23.3%), Actinomyces (13.3%), Prevotella (10%), Staphylococcus (10%), Gemella (10%), Fusobacterium (6.7%), Veillonella (6.7%), Lactobacillus (6.7%), Propionibacterium (3.3%) and Haemophilus (3.3%) | NA | NA | NA |
| Jakovljevic et al. 2018 | Molecular based | Surgical | Most common microbiome : EBV | NA | NA | NA |

CFU colony forming unit, CMP chemo mechanical preparation ,PA periapical abscess, PDT photodynamic therapy , PEI primary endodontic infection , SEI secondary endodontic infection

**Supplementary Table 6: Mean percent of different types of bacteria and viruses**

| Bacteria and viruses | N | Min | Max | Mean | SD |
| --- | --- | --- | --- | --- | --- |
| HCMV | 3 | 19% | 92% | 55.3% | 37% |
| Dialister Invisus | 3 | 33% | 68% | 51.3% | 18% |
| EBV | 3 | 14% | 78% | 51.3% | 33% |
| Micromonas Micros | 1 | 50% | 50% | 50.0% |  |
| Rodotorula Mucilaginosa | 1 | 50% | 50% | 50.0% |  |
| Campylobacter Rectus | 2 | 5% | 89% | 47.0% | 59% |
| Neisseria Spp. | 2 | 5% | 88% | 46.5% | 59% |
| P. Melaninogenica | 2 | 42% | 45% | 43.5% | 2% |
| Enterococcus Faecalis | 25 | 5% | 100% | 42.8% | 27% |
| Aggregatibacter Actinomycetemcomitans | 2 | 33% | 50% | 41.5% | 12% |
| Leptotrichia | 3 | 5% | 89% | 40.7% | 43% |
| Dialister Pneumosintes | 4 | 5% | 66% | 40.0% | 28% |
| Treponema Denticola | 12 | 12% | 56% | 38.3% | 15% |
| Candida Albicans | 5 | 27% | 47% | 37.6% | 7% |
| E. Faecium | 1 | 37% | 37% | 37.0% |  |
| Fusobacteria (Fusobacterium Spp(F. Nucleatum,Necrophorum)) | 25 | 4% | 100% | 34.5% | 29% |
| Porphyromonas Gingivalis | 20 | 4% | 100% | 33.2% | 28% |
| Propionibacterium Acnes | 2 | 23% | 42% | 32.5% | 13% |
| Porphyromonas (Endodontalis | 7 | 7% | 50% | 32.3% | 14% |
| Firmicutes | 9 | 18% | 63% | 31.0% | 14% |
| Atopobium Rimae | 2 | 10% | 50% | 30.0% | 28% |
| Prevotella Spp (Intermedia, Nigrescens, Loescheii) | 17 | 4% | 87% | 28.1% | 27% |
| Streptococcus Spp. (S. Constellatus, S. Intermedius,Streptococcus Sanguis, Salivarius, Mutans, Mitis, Oralis) | 22 | 3% | 95% | 26.0% | 25% |
| Actinomyces Naeslundii | 7 | 7% | 57% | 25.6% | 18% |
| Filifactor Alocis | 2 | 5% | 46% | 25.5% | 29% |
| Tannerella Forsythia | 10 | 3% | 57% | 25.0% | 20% |
| HSV 1 | 2 | 10% | 40% | 25.0% | 21% |
| Archae (Methanobrevibacter | 1 | 25% | 25% | 25.0% | . |
| Gemella Morbillorum | 3 | 10% | 38% | 24.3% | 14% |
| Peptostreptococcus Spp | 7 | 3% | 55% | 24.3% | 23% |
| Bacteroidetes Spp | 9 | 3% | 39% | 23.6% | 12% |
| Staphylococcus Epidermidis | 5 | 10% | 40% | 22.4% | 15% |
| Actinomyces Israelii | 3 | 13% | 32% | 22.0% | 10% |
| Proteobacteria | 7 | 9% | 46% | 21.9% | 13% |
| Lysinibacillus Fusiformis | 1 | 19% | 19% | 19.0% |  |
| Eubacterium Lentum | 3 | 6% | 28% | 18.0% | 11% |
| Actinobacteria (Actinomyces) | 8 | 6% | 57% | 17.0% | 17% |
| Capnocytophaga(Capnocytophaga Sputigena | 2 | 4% | 28% | 16.0% | 17% |
| Clostridium Bifermentans | 1 | 14% | 14% | 14.0% |  |
| Parvimonas Micra | 6 | 3% | 33% | 13.7% | 13% |
| Veillonella | 3 | 5% | 29% | 13.0% | 14% |
| Granulicatella Elegans | 1 | 10% | 10% | 10.0% |  |
| Morganella | 1 | 9% | 9% | 9.0% |  |
| Lactobacillus Rhamnosus | 3 | 6% | 10% | 8.7% | 2% |
| Lactobacillus Gasseri | 3 | 5% | 10% | 7.0% | 3% |
| Aerococcus Viridans | 2 | 6% | 8% | 7.0% | 1% |
| Burkholderia | 2 | 5% | 8% | 6.5% | 2% |
| Porphyromonas Genera | 2 | 5% | 8% | 6.5% | 2% |
| Pseudomonas Gessardii | 3 | 3% | 8% | 6.3% | 3% |
| Pseudoramibacter Alactolyticus | 3 | 2% | 8% | 5.0% | 3% |
| HPV | 1 | 5% | 5% | 5.0% |  |
| HHV-6 | 1 | 5% | 5% | 5.0% |  |
| Bifidobacterium | 2 | 4% | 5% | 4.5% | 1% |
| Synergistetes | 6 | 2% | 7% | 4.3% | 2% |
| Spirochaetes | 1 | 2% | 2% | 2.0% |  |
